# Aerobic Exercise in HIV-Associated Neurocognitive Disorders: Protocol for a Randomized Controlled Trial

**DOI:** 10.2196/29230

**Published:** 2022-01-31

**Authors:** Martins Nweke, Nombeko Mshunqane, Nalini Govender, Aderonke Akinpelu, Adesola Ogunniyi

**Affiliations:** 1 Department of Physiotherapy Faculty of Health Sciences University of Pretoria Enugu Nigeria; 2 Department of Physiotherapy Faculty of Health Sciences University of Pretoria Pretoria South Africa; 3 Department of Basic Medical Sciences Faculty of Health Sciences Durban University of Technology Durban South Africa; 4 Department of Physiotherapy Faculty of Clinical Sciences University of Ibadan Ibadan Nigeria; 5 Department of Medicine Faculty of Clinical Sciences University of Ibadan Ibadan Nigeria

**Keywords:** HIV, neurocognitive disorder, exercise, rehabilitation, quality of life, activity limitation

## Abstract

**Background:**

Since the introduction of antiretroviral therapy (ART), the incidence of HIV-associated dementia has drastically fallen. Despite using ART, people living with HIV continue to experience less severe but limiting forms of HIV-associated neurocognitive disorder (HAND). People living with HIV who are on ART and experiencing symptoms of HAND may benefit from aerobic exercise.

**Objective:**

This protocol describes a randomized controlled trial designed to determine the effects of a 12-week aerobic exercise program on HAND in Southeastern Nigeria.

**Methods:**

At least 68 patients diagnosed with HAND will be randomly placed into either an aerobic exercise group or control group. Patients in the aerobic exercise group will perform a moderate intensity workout on a stationary bicycle ergometer, 3 times a week for 12 weeks. We will measure the primary outcomes including neurocognitive performance, prevalence of HAND, viral load, and CD4 count. We will evaluate postexercise neurocognitive performance using reliable neuropsychological tests relevant to people living with HIV, in line with the Frascati criteria. We will assess secondary outcomes such as quality of life, activity limitation, and social participation using the World Health Organization Quality of Life (WHOQOL)-Brief, and the Oxford Participation and Activities questionnaire. We will use exploratory statistics to test the data for normality and homogeneity. We will analyze the effect of the exercise program on HAND using relative risk (RR) and absolute risk reduction (number needed to treat). Analysis of covariance will be run to estimate the effect of exercise on quality of life and activity and participation level.

**Results:**

This funded trial was approved by the Institutional Review Board in May 2020. The protocol was approved on June 15, 2020. Enrollment commenced in January 2021 and was completed in May 2021. Over 60% of the participants were recruited at the time of first submission to JMIR Mental Health. Data curation is still ongoing; hence, data analysis is yet to be executed. Study outcomes are expected to be published in March 2022.

**Conclusions:**

This is a protocol for a randomized controlled trial that aims to evaluate the effect of a 12-week aerobic exercise program on HAND in Southeastern Nigeria.

**Trial Registration:**

Pan African Clinical Trials Registry PACTR202009483415745; https://tinyurl.com/2p97zpu9

**International Registered Report Identifier (IRRID):**

PRR1-10.2196/29230

## Introduction

### Background

HIV-associated neurocognitive disorder (HAND) is a common neurological complication reported among people living with HIV [1]. Before antiretroviral therapy (ART) was introduced in 1996, HIV-associated dementia was a progressive disorder leading to death within 6 months [2]. Since the introduction of ART, the mean survival rate following HIV-associated dementia has increased, and milder forms of HAND have become more prevalent [1,3-5]. Globally, approximately 50% of people living with HIV are affected by HAND, with rates varying across countries [6-8]. In resource-constrained African settings, the burden of HAND ranges from 14% to 88.3% [1,9,10] in contrast with 19% to 52% in resource-limited countries [11,12]. In sub-Saharan Africa, HAND affects between 18.8% and 88.3% of people living with HIV, with a pooled prevalence of 53% [13]. In Nigeria, the prevalence of HAND fluctuates with ART use and lies between 21.5% and 71.7% [1,14,15]. People living with HIV who have HAND often present with cognitive impairment as well as behavioral and motor abnormalities such as memory loss, impulsiveness, irritability, visuospatial difficulty, dyscalculia, and difficulty with concentration and attention [2,3]. Impaired cognitive ability impacts quality of life (QoL) and treatment adherence [16]. People with HAND may also progress from being asymptomatic to being severely impaired [17,18]. People with HAND generally have limited functional capacity resulting in low productivity, job loss, poverty, poor academic performance, reduced QoL, and poor treatment adherence [19,20].

Global efforts directed at eradicating HAND [20] include early intensification of ART [21] and use of intranasal insulin [22], psychostimulants [23], and adjunctive therapies [21,24]. According to current guidelines, ART should start as soon an individual is diagnosed with HIV with a cluster of differentiation-4 (CD4) count ≤500 cells/mm^3^ [25]. Timely ART initiation has led to a marked decline in the incidence of HIV-associated dementia. Although severe forms of HAND have become less common, people living with HIV continue to experience less severe but limiting forms of HAND despite ART use. The increasing incidence of HAND may be due to early HIV entry into the central nervous system, limited permeability of ART through the blood-brain barrier (BBB), reduced ART efficacy, increased drug resistance, virologic failure, adverse effects, and neurotoxicity [26-29].

A recent scoping review revealed limited rehabilitative treatment options for HAND [30]. Rehabilitation may include psychocognitive training [31] and physical exercise [32]. Psychocognitive exercises involving pen-and-paper or computerized cognitive programs are based mainly on restoring cognitive function [28,33]. These interventions often include cognitive training, cognitive stimulation, and cognitive rehabilitation using different tasks [28]. Examples include Captain’s log [34], Smart-Brain [35], and InSight [36]. In contrast, physical exercise interventions are compensatory and have been shown to slow down the progression of cognitive disorder in aging HIV-seronegative individuals [31,37-39]. Currently, few exercise interventions and treatment guidelines exist for rehabilitating HAND except for evidence-informed recommendations reported by O’Brien et al [40]. Although physical exercise may slow the decline in cognitive functioning among people living with HIV, research-generated evidence remains inconclusive due to heterogeneity in study designs and use of low-intensity exercises [32,41,42]. A recent systematic review revealed that the effect of structured exercise interventions on cognitive performance of individuals with HAND has not been investigated [43]. In HIV-negative individuals, long-term and intense aerobic exercise improves BBB permeability, enhances synaptic plasticity, improves neurotrophin secretion, and regulates neuroinflammation [15,44,45] and thus may benefit people with HAND. This study therefore aims to determine the effect of a 12-week aerobic exercise program on HAND. This protocol describes procedures that will be implemented to determine the effect of a 12-week aerobic exercise program on HAND. The data will provide supporting evidence about the suitability of aerobic exercise as a complementary therapy for mitigating neurocognitive disorder among people living with HIV. The outcomes will also provide evidence to strengthen the advocacy for including aerobic exercise in the management of people living with HIV experiencing neurocognitive disorder.

### Objectives

The specific objectives of this study are to determine the effect of a 12-week aerobic exercise program on the severity of HAND symptoms, determine the effect of a 12-week aerobic exercise program on CD4 count in individuals with HAND, determine the effect of a 12-week aerobic exercise program on plasma viral load in individuals with HAND, determine the effect of a 12-week aerobic exercise program on functional activity and social participation of individuals with HAND, and determine the effect of a 12-week aerobic exercise program on QoL of individuals with HAND.

### Trial Design

This is a parallel randomized controlled trial employing a restricted assignment scheme, where participants were allocated in a 1:1 ratio. The intervention is aerobic exercise, and the comparator is a no-treatment control group. All assessors will be blinded regarding participant identification of both the experimental and control groups.

## Methods

### Study Setting

This study is taking place at the Exercise Immunology Clinic of the Department of Physiotherapy, University of Nigeria Teaching Hospitals (UNTH) Ituku-Ozalla, Nigeria, and the University of Nigeria Enugu Campus (UNEC). A preliminary study revealed that approximately 50% of the prospective participants that visited the UNTH ART clinic were from Enugu Metropolis. The second site, UNEC, was chosen as a more centralized location for participants who resided in Enugu Metropolis and nearby environs. Participants were purposively selected to participate. To ensure consistency, the intervention team, which is comprised of 2 qualified physiotherapists and 2 trained research assistants, was trained by the principal investigator.

We identified prospective participants during a pilot study. Prospective participants, who lived in the Enugu metropolis and surrounds, were invited by text message to attend the ART clinic. Only participants able to travel to the study site with ease were invited to participate. Participants were randomly assigned to the intervention or control group. First, a sequence of random numbers was generated using Random Restricted Software 2.0. An independent person assigned the random numbers to either the intervention or control group by placing the generated numbers into A4, opaque, sealed envelopes, with only C or E written on an inconspicuous area of the envelope. Envelopes with C are control and E are exercise. The outcome assessors (the principal investigator and clinical psychologist) enrolled participants into the study, without knowing group assignments. Outcome assessors, including the principal investigator, neurologist and clinical psychologist, and data analyst, were also blinded while conducting neurological assessments. Care physicians were asked not to suggest any form of aerobic exercise to the patients throughout the study period. Trained physiotherapists conducted the treatment. Finally, the data were coded (C for control group and B for experimental group) so that the biostatistician will not know which group is experimental or control.

### Eligibility Criteria

We included patients if they met the following criteria: diagnosed with HAND and physically inactive (sedentary, <2 hours of exercise per week; ready to exercise upon assessment, not engaged in regular exercise for approximately 3 months before the study). Patients were excluded if they were older than 65 years; had uncontrolled hypertension (blood pressure [BP] ˃140/90 mm Hg), deafness, severe eye impairment, physical disability, history of traumatic brain injury, psychiatric illness, recent focal neurological deficit, active depression, alcohol or substance abuse, musculoskeletal injury, or acute illness capable of hampering exercise performance; pregnancy; or had angina pectoralis and/or shortness of breath at rest or during exercise. We excluded participants on cognition-enhancing drugs such as eugeroics, attention deficit/hyperactive disorder medications, and nootropic supplements.

### Informed Consent

Informed written consent was obtained from each participant before enrollment in the study, provided they had the capacity to give consent.

In this study, the control group receives no treatment. The efficacy of aerobic exercise in HAND rehabilitation has rarely been investigated; therefore, we are comparing aerobic exercise to no exercise, before comparing to other forms of exercise or therapy.

### Exercise Testing

Exercise testing is conducted using the Young Men Christian Association (YMCA) bicycle ergometer protocol at baseline and after a 12-week exercise program [46,47]. The YMCA protocol uses 2 to 4 stages of continuous exercise lasting 3 minutes, during which 2 heart rate (HR)-power output data points (steady-state HR) between 110 bpm and 150 bpm are needed. The test is designed to raise the participant’s steady-state HR to between 110 bpm and 150 bpm and 70% HR reserve or 85% of the age-predicted maximum HR (HRmax) for at least 2 consecutive stages. Using the Life-Fitness Cycle Ergometer (95Ci, Franklin Park, IL), the first 3-minute workload is set between 150 kg·m·min^-1^ and 300 kg·m·min^-1^ (25-50 watts). The speed is set at 50 rpm. HR is measured within the last minute of each stage. If an HR >110 bpm is obtained in the first 3 minutes, then only one additional 3-minute stage is performed by increasing the workload to 450 kg·m·min^-1^ (75 watts). If the second-stage HR is <110 bpm, the 3-minute third or fourth stage is performed at an additional workload of 150 kg·m·min^-1^ up to 750 kg·m·min^-1^ (125 watts), in order to obtain 2 HRs between 110 bpm and 150 bpm. At the end of the test, a 3-minute recovery period (cool down) at zero resistance is administered. HR is measured during the last minute of each stage. The 2 steady-state HRs are plotted against the respective workload on the YMCA graph sheet. The line generated from the plotted points is then extrapolated to the age-predicted HRmax, and a perpendicular line is dropped to the x axis to estimate the work rate (VO_2max_) that would have been achieved if the individual had worked to maximum capacity [46-48]. At the end of exercise testing, the participants are asked to return to the Physiotherapy department within 2 days to 3 days to commence the intervention.

### Exercise Intervention

Participants in the aerobic exercise group exercise on a bicycle ergometer at a low intensity of between 60% and 80% of their HRmax as recommended by the American College of Sports Medicine (ACSM) [49]. Participants train 3 times a week for 12 weeks. Initially, participants train at 60% of HRmax, and this is increased after 4 weeks to 80% HR_max_ for the remainder of the training period. Each training session consists of 20 minutes to 30 minutes of aerobic exercise in the first 4 weeks depending on the patient’s tolerance. After the first 4 weeks, training sessions are increased to 30 minutes to 45 minutes and further increased after the eighth week to 60 minutes for the remainder of the intervention. Participants are encouraged to give their best to the moderate-intensity exercise. Participants are prepared for exercise following the ACSM guidelines [46]. All fitness testing is performed by qualified physiotherapists.

### Control Group

Participants are educated on the benefits of exercise for people living with HIV but are asked not to engage in any form of structured physical activity for the corresponding 12-week period. The first education session occurs while the exercise participants are being moved to the trial site, which serves to distract the control group participants. The second education session takes place 6 weeks into the intervention, during which participants are asked if they have engaged in any structured physical activity and if yes, they are asked to quantify the intensity and time. We encourage control group participants to abstain from structured physical activity.

### Criteria for Discontinuing or Modifying Allocated Interventions

The aerobic exercise intervention is discontinued or modified if participants experience exercise-related angina pectoralis or shortness of breath during 2 successive sessions, exercise-induced tachycardia during an exercise session, severe illness capable of affecting the participant’s exercise capacity, complaints of worsening cognitive ability, or if participants request to discontinue or modify the exercise intensity.

### Strategies to Improve Adherence to Interventions

During the pilot study, we noted that one of the major challenges faced by our prospective participants was increased transportation costs and the attendant opportunistic costs of participants who will not be able to work due to the study. Participants are given a sum of N2000 (US $4.86) every 2 weeks to cover transport costs. We call participants on the day before their exercise session to remind them of their appointment. Participants are called by telephone if they fail to show up for training or a periodic appointment to ascertain the reason for their absence and improve compliance.

### Relevant Concomitant Care Permitted or Prohibited During the Trial

Participants continue with their ART. Participants are discouraged from continuing any medication not prescribed by a physician. Prospective participants are allowed a washout period of 2 weeks before being eligible to continue.

### Study Outcomes

The primary outcomes include neurocognitive performance, prevalence of HAND, viral load, and CD4 count. The secondary outcomes include maximum oxygen uptake (VO_2_), QoL, activity limitation, and participation restriction. Potential confounding variables include age, sex, level of education, vaccination, history of virologic failure, level of ART adherence, exercise adherence, ART regimen, ovulation status, history of recent vaccination, and seasonality. These variables will be measured at baseline, after 12 weeks, and 3 months after the intervention. Their change will be measured over time. Aggregation parameters will include proportion, mean, or median depending on how the data are distributed.

### Participant Timeline

**Figure 1:**
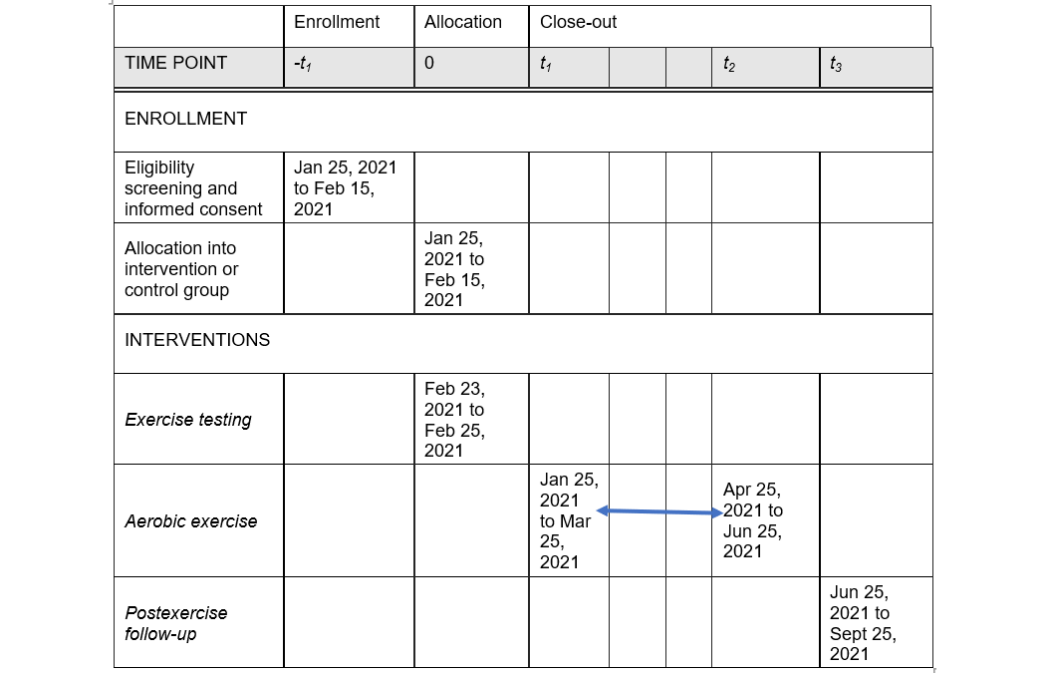
Proposed timeline for the randomized controlled trial for measuring the efficacy of exercise for rehabilitating symptoms associated with HIV-associated neurocognitive disorder (HAND) in people living with HIV.

The proposed timeline for the study and planned elements is shown in Figure 1. All prospective participants were identified in a pilot study. Baseline assessments were conducted from the end of January 2021 to mid-February 2021 and covered neurocognitive performance, BP, HR, respiratory rate, assessment of physical activity readiness, QoL, CD4 count, viral load, and activity limitation and social participation. Before the intervention, all participants undergo an exercise stress test. The aerobic exercise intervention starts a day after exercise testing and lasts for 12 weeks. Following the 12-week aerobic intervention, postexercise assessments are conducted.

### Sample Size

An estimated sample size of 68 (34 in each group) will have 90% power to detect a difference in means of 13.4 (the difference between a Group 1 mean, µ_1_, of 56 and a Group 2 mean, µ_2_, of 42.6) assuming that the common SD is 16.67 using a 2-group *t* test with a 5% 2-sided significance level.

### Neuropsychological Screening

The principal investigator (who is a physiotherapist working with people living with HIV and neurological conditions), a clinical psychologist, and a neurologist (who is a specialized medical doctor) conduct the neuropsychological screening, which is conducted in 3 stages. First, we conduct a brief neuromedical screening using a pilot assessment guide; then, we administer the neuropsychological instruments and, finally, assess the subjective symptoms of HAND such as difficulty remembering recent events (people, conversations, names, commitments, where things are placed), understanding conversation or reading materials, word finding, planning activities, problem solving, concentrating, thinking clearly or logically, finding his or her way about, calculating, and following direction or instruction.

We administer neuropsychological instruments chosen for their simplicity and ease of administration in any language. Only the Hopkins Verbal Learning Test-Revised (HVLT-R) and Controlled Oral Word Association Test (COWAT) require understanding of some English words. These tests were extracted from the international neurobehavioral test battery used by the HIV Neurobehavioral Research Center [1] and a recent clinical trial on HAND [50,51]. These tests are sensitive to HAND in Nigeria [52,53]. We first screen for probable dementia using the International HIV Dementia Scale (IHDS). Confirmatory neuropsychological tests are administered in the following order: first, we administer the HVLT-R immediate recall (duration 3-5 minutes). After waiting 20 minutes to 25 minutes to administer the second part of the HVLT-R, we administer the Trail Making Test (TMT)-A and -B (5-10 minutes), verbal fluency (3-8 minutes), and the Digit Span Test (5-10 minutes). We then administer the HVLT-R delay recall. We also assess neurocognitive performance in line with the 2007 modified American Academy of Neurology criteria, also known as the Frascati criteria [1,54]. We will convert the raw scale scores using a clinical rating algorithm, to sum the scores to obtain an overall score for each participant. The latter will be used for covariate analysis, if needed.

### Tests

#### Beck Depression Inventory

The Beck Depression Inventory (BDI) [55] measures characteristic attitudes and symptoms of depression using a 21-item self-report rating inventory (Multimedia Appendix 1). The BDI takes approximately 10 minutes to complete and requires a fifth- to sixth-grade reading level to adequately understand the questions. Internal consistency ranges from 0.73 to 0.92, with a mean of 0.86 [56]. The BDI has demonstrated high internal consistency, with alpha coefficients of 0.86 and 0.81 for psychiatric and nonpsychiatric populations, respectively [57]. A score ≥17 indicates borderline clinical depression.

#### Alcohol Use Disorder Identification Test

The Alcohol Use Disorder Identification Test (AUDIT) [58] is approved by the World Health Organization (WHO) to assess intoxication or withdrawal (Multimedia Appendix 2). The AUDIT is comprised of 10 items, and a score ≥8 indicates alcohol intoxication or withdrawal. Patients with scores >8 were excluded from the study [12]. It takes 2 minutes to 4 minutes to complete.

#### Drug Abuse Screening Test

The Drug Abuse Screening Test [59] is a valid and reliable instrument consisting of 10 items (Multimedia Appendix 3). Patients who score ≥3 are suspected of drug abuse and were excluded from the study. It takes approximately 5 minutes to administer.

#### International HIV Dementia Scale

We screen HIV-positive patients for dementia and cognitive impairment using the IHDS [60] (Multimedia Appendix 4). The IHDS tests registration, recall, motor function, and information processing. The IHDS has a sensitivity and specificity of 74% and 46%, respectively, at a cutoff point of 9.5. The test does not require any special instruments except a timer or wristwatch and can be easily administered by other health workers, not necessarily by a physician. The IHDS is also free of cultural bias and can be used in many resource-limited countries.

#### Controlled Oral Word Association Test

We use the COWAT [61] to assess verbal fluency using FAS letter fluency—number of words generated (Multimedia Appendix 5). Verbal fluency measures cognitive function that facilitates information retrieval from memory, and the verbal fluency test evaluates an individual's ability to retrieve specific information within restricted search parameters [62]. This test requires the individual to name as many words as possible that begin with a given letter (ie, F, A, and S). Each letter is allotted 60 seconds. Individuals cannot use proper names or numbers and cannot use words with different tenses or endings once the root word has been given. They have to do it as quickly as possible, and the number of words produced during 1 minute is scored for both phonemic and semantic verbal fluency [62]. The test takes 3 minutes to 8 minutes to complete. The score equals the mean number of words uttered in the 3 trials corresponding to each initial letter [63]. This test does not require special instrumentation.

#### Hopkins Verbal Learning Test-Revised

The HVLT-R [64] is used to assess verbal learning and memory or recall (Multimedia Appendix 6). The HVLT-R is simple to administer and is similar to the California Verbal Learning Test [65]. An assessor gives the patient a list of 12 words with an embedded semantic structure (4 categories of 3 words each). The assessor reads the list to the patient, who is then asked to repeat as many words as possible in any order (free recall). This process is repeated 3 times, which represents the 3 learning trials. After a 25-minute break, the patient is again asked to remember as many of the words as possible in any order. The patient’s semantic strategy is evaluated by examining the degree to which words are semantically clustered during the 3 learning trials. In the standard administration, items from the same category are not presented together, and subjects are not informed of the semantic organization. The HVLT-R’s 3 learning trials and delay recall trial are scored separately. The 3 learning trial scores (number of correct words) are summed to yield a total score. Overall, this test takes 28 minutes to 30 minutes. This test does not require instrumentation.

#### Trail Making Test-A and -B

The TMT is a 2-in-1, sensitive, paper-and-pencil measure of information processing speed and executive function [66,67] (Multimedia Appendix 7). The TMT consists of 2 parts (TMT-A and TMT-B). The TMT-A consists of a standardized page on which the numbers 1 to 25 are scattered within circles, and the participants are asked to connect the numbers in order as quickly as possible. Similarly, the TMT-B consists of a standardized page that includes the numbers 1 to 13 and the letters A to L. The participants are instructed to draw lines connecting numbers and letters in order, alternating numbers and letters. Before starting the test, participants are allowed to practice on 6 items to make sure that they understand both tasks. When a participant makes an error during the test, the examiner points it out, explains, and then guides the participant to correctly complete the circles, after which the participants are requested to continue with the task. A maximum time of 300 seconds is allowed before discontinuing the test. Direct scores of TMT will be the time in seconds taken to complete each task (-A and -B). This test takes 5 minutes to 10 minutes.

#### Digit Span Test

The digit span test (DST) [68] is a pencil-and-paper instrument and evaluates auditory attentional capacity and working memory for orally presented information (Multimedia Appendix 8). In this study, the DST is used to assess attention and working memory. The DST was originally developed for people between 18 years and 97 years old and is appropriate for use in this study. Participants are asked to repeat series of digits that become gradually longer. The maximum digit span that the participants are able to repeat in direct and reverse orders constitutes the forward (DST-f) and backward (DST-b) scores, respectively [51,68]. This test should be completed in 10 minutes to 15 minutes.

#### The Lawton Instrumental Activities of Daily Living Scale

The Lawton Instrumental Activities of Daily Living Scale is a valid and sensitive measure of instrumental activities of daily living and is comprised of 8 items (Multimedia Appendix 9). Scores <8 may indicate functional impairment [9]. This test takes 3 minutes to 5 minutes to complete.

#### The WHO Quality of Life-BREF

We use the short form of the World Health Organization Quality of Life (WHOQOL)-BREF, which has been validated in diverse settings, including African countries, and is based on a well-classified definition of QoL (Multimedia Appendix 10). It is comprised of physical, psychological, social, and environment domains. The WHOQOL-BREF is a recommended instrument for people living with HIV infection [69,70]. The WHOQOL-BREF has an internal consistency of α=0.74-0.85 and test-retest reliability of rho=0.64-0.79 [71]. Each of the 4 domains is measured on a 5-point Likert scale: 1 indicates low perception, and 5 indicates high perception [69]. The WHOQOL-BREF measures the perceived QoL and hence contains items asking how patients felt about different facets of life in the week before being assessed.

#### The Oxford Participation and Activities Questionnaire

The Oxford Participation and Activities Questionnaire (Ox-PAQ) is a 23-item, generic, patient-reported outcome measure (Multimedia Appendix 11). Theoretically, it is grounded in the WHO International Classification of Functioning, Disability and Health [72]. It is primarily used in clinical trials to evaluate interventions targeted at improving or maintaining participation and activity. The measure demonstrates good reliability (Cronbach α=0.81-0.96) and validity and low levels of missing data across all 3 domains [73,74]. It equally demonstrates good convergent validity with the EuroQol-5D questionnaire [75].

#### Physical Activity Readiness Questionnaire

The Physical Activity Readiness Questionnaire (PAR-Q) was created by the British Columbia Ministry of Health and the Multidisciplinary Board on Exercise [76] (Multimedia Appendix 12). It is a simple self-screening tool that is used to plan an exercise program. The tool helps to determine the readiness for exercise as it reveals the safety or possible risk of exercising for an individual based on their health history, current symptoms, and risk factors. It is often used in clinical trials to ascertain readiness prior to enrollment [77].

#### Cardiorespiratory Measurements

Participants’ resting HR, systolic BP, and diastolic BP are monitored on the right arm [46,78] using an automated digital electronic BP monitor (Omron digital BP monitor, Model M2 Eco; Tokyo, Japan). These measurements are monitored between 7:00 am and 2:00 pm each test day.

#### Anthropometric Measurements

We assess participants’ physical characteristics (% body fat, weight in kg, height in meters, and BMI in kg/m^2^) according to a standardized anthropometric protocol [79,80].

#### Blood Sample Collection

Blood samples are collected using the venipuncture method. We collect venous blood samples both pre- and posttreatment between 8:00 am and 12:00 pm. We collect blood samples using a 5-mL syringe [48]. CD4 count tests are conducted within 12 hours, and samples for viral load are stored in a refrigerator at –80 °C until analysis [81].

#### Measurement of CD4 Count and Viral Load

Samples are analyzed by the UNTH ART clinic laboratory scientist. To control for the potential effects of rest, time of the day, season, ovulation, and vaccination, pre-exercise blood samples for quantifying CD4 count were drawn when patients arrived at the laboratory, after 60 minutes of rest [82]. To minimize diurnal variation, samples were collected between 8:00 am and 12:30 pm. We aimed to collect pre-exercise blood samples before the heavy rainfall season in June. In Nigeria, rainfall peaks in June [83] and is associated with increased opportunistic infections that influence CD4 count. After each sample was collected, we examined the tube for integrity before transporting to the testing center. All CD4 counts were measured within 12 hours of sample collection following the recommendation of the WHO [84-86].

### Data Management

Data are manually transcribed from paper forms into a Microsoft Excel spreadsheet and exported and secured to MicrOsiris 24.8. Data are verified through independent double data entry, where the principal investigator (data manager) and a data clerk both enter data. Consistency checks are performed during data entry, and warnings are displayed when needed.

Personal information such as contact number and identity number are collected and only used to reach participants when necessary and for possible access to participants’ hospital files. Data are handled confidentially and are not shared with a third party. Participants’ names do not appear in any data record except in a case of referral.

### Statistical Methods

Primary and secondary variables will be tested for normality and heterogeneity using Kolmogorov-Smirnov and Levene tests, respectively. We will compare the control group and exercise group using a 2-way analysis of variance with repeated measurements and Bonferroni correction. Primary outcome measures are cognitive performance, QoL, activity limitation, and participation. We will compute hazard ratios to evaluate the effect of the intervention over time. SPSS version 21 (IBM Corp, Armonk, NY) will be used.

We expect that potential confounding variables, not accounted for by randomization, may influence outcomes between the control and study groups. We will include potential confounding variables in appropriate analysis of covariance. Covariates include neurocognitive performance test scores (clinical rating algorithm), CD4 count (classified as <350 cells/µl or ≥350 cells/µl), viral load (classified as detectable [>400 copies/mL] or undetectable [<400 copies/mL]), age, sex, level of education, BMI, exercise adherence (classified as nonadherent [≤40%] or adherent [>40%]), adherence to ART since the intervention (classified as nonadherent [≤40%] or adherent [>40%]), ART regimen, ovulation, and vaccination status. Also, our findings will be included in an updated meta-analysis of the effects of exercise on cognition in people living with HIV to understand how the study outcome may drive existing associations. The initial meta-analysis was conducted by our team [43].

As per nonadherence to a study protocol, we will employ intention-to-treat analysis. Effort will be made to prevent missing data through cross-checking of the information obtained from participants. In case of missing data, we will explore patterns of missing and, where appropriate, multiple computation will be executed using SPSS version 21.

### Plans to Give Access to the Full Protocol, Participant-Level Data, and Statistical Code

This decision is subject to approval by the Physiotherapy Department of the University of Pretoria, South Africa.

### Oversight and Monitoring

The trial is conducted by a team of 2 qualified physiotherapists and 2 trained research assistants, while the investigator and research supervisors monitor and oversee data collection and analysis. The intervention administrator provides daily updates regarding the trial to the chief investigator who then provides weekly updates to his supervisors. Challenges encountered during the trial are resolved by the investigators through conference meetings or other feasible alternatives.

### Adverse Event Reporting and Harms

Participants are asked to report any adverse event following exercise. Adverse events are formally assessed every 2 weeks using an adverse event form piloted by the US Medical Device and Diagnostic Industry recommendations [87,88] (Multimedia Appendix 13). If an adverse event is reported, the patient is referred to their physician for immediate assessment of underlying cause and possible management. A physiotherapist treats cases of pain, lower back pain, fatigue, and muscle soreness and prescribes rest to the participants when necessary. In cases of spontaneous but mild adverse events, patients are given sufficient time to rest, after which a therapist decides if the participant is fit to continue the scheduled exercise.

### Plans for Communicating Important Protocol Amendments to Relevant Parties

Amendments to the trial protocol with respect to eligibility criteria, outcomes, analysis, and frequency and duration of treatment will be communicated first to the researchers’ supervisors and then the University of Pretoria, Faculty of Health Sciences Research Ethics Committee, the PAN Africa Trial Registry, and the journal in which the protocol is published.

### Dissemination Plans

The outcome of the trial will be communicated to participants and health care professionals through conference presentations and to the general public through publication in a peer-reviewed international journal. No publication restriction applies. The data will be available for sharing upon request, which is subject to approval by the Department of Physiotherapy, University of Pretoria.

### Ethics Approval

Ethical approval was obtained from the University of Pretoria research ethics committee (Ethics reference no. 152/2020). Informed consent was obtained before enrollment. Prior to consent seeking, we introduced the study and explained the purpose thereof. Participants reserved the right to make decisions regarding their participation without inducement and such right was upheld throughout the study.

## Results

The trial, which secured funding in March 2020, was approved by the Institutional Review Board in May 2020. Data collection commenced in June 2020, with a pilot study to examine the rater reliability and minimum detectable change of the selected neuropsychological tests. Between July 2020 and November 2020, individuals with HAND had been identified. Participant enrollment commenced in January 2021 and was completed in May 2021. An amendment was submitted and secured ethical approval. Over 60% of the participants were recruited at the time of first submission to JMIR Mental Health. Data curation is still ongoing; hence, data analysis is yet to be executed. Study outcomes are expected to be published in March 2022.

## Discussion

In line with the ART clinic’s COVID-19 prevention guidelines, personal protective equipment including face masks and hand sanitizer are used by research team members and participants, while ensuring social distancing. When considering the acceptability of incentives in clinical trials, evidence suggests that incentives may ensure a good degree of adherence and completion [89-92]. Several systematic reviews [90-92] have argued that incentives cover opportunity costs of participating in behavioral interventions such as exercise. Participants will be compensated for transport money to the hospital only.

## Appendix

Multimedia Appendix 1 Beck Depression Inventory.

Multimedia Appendix 2 Alcohol Use Disorder Identification Test (AUDIT).

Multimedia Appendix 3 Drug Abuse Screening Test.

Multimedia Appendix 4 International HIV Dementia Scale.

Multimedia Appendix 5 Controlled Oral Word Association Test (COWAT) (Verbal Fluency Test).

Multimedia Appendix 6 Hopkin Verbal Learning Test-Revised.

Multimedia Appendix 7 Trail Making Test A & B.

Multimedia Appendix 8 Digit span test.

Multimedia Appendix 9 Lawton Instrumental Activities of Daily Living (IADL) scale.

Multimedia Appendix 10 The World Health Organization Quality of Life (WHOQoL) Bref.

Multimedia Appendix 11 Oxford Participation and Activities Questionnaire (Ox-PAQ).

Multimedia Appendix 12 Physical Activity Readiness Questionnaire.

Multimedia Appendix 13 Adverse events form.

## Abbreviations

ACSM: American College of Sports Medicine
ART: antiretroviral therapy
AUDIT: Alcohol Use Disorder Identification Test
BBB: blood-brain barrier
BDI: Beck Depression Inventory
BP: blood pressure
CD4: cluster of differentiation-4
COWAT: Controlled Oral Word Association Test
DST: digit span test
HAND: HIV-associated neurocognitive disorder
HR: heart rate
HRmax: age-predicted maximum HR
HVLT-R: Hopkins Verbal Learning Test-Revised
IHDS: International HIV Dementia Scale
NSFAS: National Student Financial Aid Scheme
Ox-PAQ: Oxford Participation and Activities Questionnaire
QoL: quality of life
TMT: Trail Making Test
UNEC: University of Nigeria Enugu Campus
UNTH: University of Nigeria Teaching Hospitals
VO_2_: maximum oxygen uptake
WHO: World Health Organization
WHOQOL: World Health Organization Quality of Life
YMCA: Young Men Christian Association

## References

[1] Yakasai AM, Gudaji MI, Muhammad H, Ibrahim A, Owolabi LF, Ibrahim DA, Babashani M, Mijinyawa MS, Borodo MM, Ogun AS, Habib AG (2015). Prevalence and correlates of HIV-associated neurocognitive disorders (HAND) in Northwestern Nigeria. Neurol Res Int.

[2] Clifford DB, Ances BM (2013). HIV-associated neurocognitive disorder. The Lancet Infectious Diseases.

[3] Sacktor N, Skolasky RL, Seaberg E, Munro C, Becker JT, Martin E, Ragin A, Levine A, Miller E (2015). Prevalence of HIV-associated neurocognitive disorders in the Multicenter AIDS Cohort Study. Neurology.

[4] Heaton RK, Clifford DB, Franklin DR, Woods SP, Ake C, Vaida F, Ellis RJ, Letendre SL, Marcotte TD, Atkinson JH, Rivera-Mindt M, Vigil OR, Taylor MJ, Collier AC, Marra CM, Gelman BB, McArthur JC, Morgello S, Simpson DM, McCutchan JA, Abramson I, Gamst A, Fennema-Notestine C, Jernigan TL, Wong J, Grant I (2010). HIV-associated neurocognitive disorders persist in the era of potent antiretroviral therapy: CHARTER Study. Neurology.

[5] Heaton R, Franklin D, Ellis R, McCutchan J, Letendre S, Leblanc S, Corkran SH, Duarte NA, Clifford DB, Woods SP, Collier AC, Marra CM, Morgello S, Mindt MR, Taylor MJ, Marcotte TD, Atkinson JH, Wolfson T, Gelman BB, McArthur JC, Simpson DM, Abramson I, Gamst A, Fennema-Notestine C, Jernigan TL, Wong J, Grant I, CHARTER Group, HNRC Group (2011). HIV-associated neurocognitive disorders before and during the era of combination antiretroviral therapy: differences in rates, nature, and predictors. J Neurovirol.

[6] Clifford D (2017). HIV-associated neurocognitive disorder. Curr Opin Infect Dis.

[7] Cysique LA, Heaton RK, Kamminga J, Lane T, Gates TM, Moore DM, Hubner E, Carr A, Brew BJ (2014). HIV-associated neurocognitive disorder in Australia: a case of a high-functioning and optimally treated cohort and implications for international neuroHIV research. J Neurovirol.

[8] Robertson K, Bayon C, Molina J, McNamara P, Resch C, Muñoz-Moreno Jose A, Kulasegaram R, Schewe K, Burgos-Ramirez A, De Alvaro C, Cabrero E, Guion M, Norton M, van Wyk J (2014). Screening for neurocognitive impairment, depression, and anxiety in HIV-infected patients in Western Europe and Canada. AIDS Care.

[9] Lawler K, Mosepele M, Ratcliffe S, Seloilwe E, Steele K, Nthobatsang R, Steenhoff A (2010). Neurocognitive impairment among HIV-positive individuals in Botswana: a pilot study. J Int AIDS Soc.

[10] Atashili J, Gaynes BN, Pence BW, Tayong G, Kats D, O'donnell JK, Ndumbe PM, Njamnshi AK (2013). Prevalence, characteristics and correlates of a positive-dementia screen in patients on antiretroviral therapy in Bamenda, Cameroon: a cross-sectional study. BMC Neurol.

[11] Van Wijk C (2013). Screening for HIV-associated neurocognitive disorders (HANDs) in South Africa: A caution against uncritical use of comparative data from other developing countries. South. Afr. j. HIV med.

[12] Yusuf AJ, Hassan A, Mamman AI, Muktar HM, Suleiman AM, Baiyewu O (2017). Prevalence of HIV-associated neurocognitive disorder (HAND) among patients attending a tertiary health facility in Northern Nigeria. J Int Assoc Provid AIDS Care.

[13] Nweke MC, Okemuo AJ, Uduonu EM, Ugwu PI, Nwachukwu C, Mshunqane N (2021). Meta-analysis of factors affecting prevalence estimates of HIV-associated neurocognitive disorder in sub-Saharan Africa. S. Afr. J. Sci.

[14] Kelly CM, van Oosterhout JJ, Ngwalo C, Stewart RC, Benjamin L, Robertson KR, Khoo S, Allain TJ, Solomon T (2014). HIV associated neurocognitive disorders (HAND) in Malawian adults and effect on adherence to combination anti-retroviral therapy: a cross sectional study. PLoS One.

[15] Modi G, Mochan A, Modi M (2018). Neurological manifestations of HIV. Advances in HIV and AIDS Control.

[16] Morgan EE, Woods SP, Grant I, HIV Neurobehavioral Research Program (HNRP) Group (2012). Intra-individual neurocognitive variability confers risk of dependence in activities of daily living among HIV-seropositive individuals without HIV-associated neurocognitive disorders. Arch Clin Neuropsychol.

[17] Grant I, Franklin DR, Deutsch R, Woods SP, Vaida F, Ellis RJ, Letendre SL, Marcotte TD, Atkinson JH, Collier AC, Marra CM, Clifford DB, Gelman BB, McArthur JC, Morgello S, Simpson DM, McCutchan JA, Abramson I, Gamst A, Fennema-Notestine C, Smith DM, Heaton RK (2014). Asymptomatic HIV-associated neurocognitive impairment increases risk for symptomatic decline. Neurology.

[18] Alford K, Vera J (2018). Cognitive impairment in people living with HIV in the ART era: a review. Br Med Bull.

[19] Singh D, Joska JA, Goodkin K, Lopez E, Myer L, Paul RH, John S, Sunpath H (2010). Normative scores for a brief neuropsychological battery for the detection of HIV-associated neurocognitive disorder (HAND) among South Africans. BMC Res Notes.

[20] Bougea A, Spantideas N, Galanis P, Gkekas G, Thomaides T (2019). Optimal treatment of HIV-associated neurocognitive disorders: myths and reality. A critical review. Ther Adv Infect Dis.

[21] Figuera-Losada M, Stathis M, Dorskind JM, Thomas AG, Bandaru VVR, Yoo S, Westwood NJ, Rogers GW, McArthur JC, Haughey NJ, Slusher BS, Rojas C (2015). Cambinol, a novel inhibitor of neutral sphingomyelinase 2 shows neuroprotective properties. PLoS One.

[22] Singer EA, Thames AD (2016). Neurobehavioral manifestations of human immunodeficiency virus/AIDS: diagnosis and treatment. Neurol Clin.

[23] McGuire JL, Barrett JS, Vezina HE, Spitsin S, Douglas SD (2014). Adjuvant therapies for HIV-associated neurocognitive disorders. Ann Clin Transl Neurol.

[24] Lewden C, Bouteloup V, De Wit S, Sabin C, Mocroft A, Wasmuth JC, van Sighem A, Kirk O, Obel N, Panos G, Ghosn J, Dabis F, Mary-Krause M, Leport C, Perez-Hoyos S, Sobrino-Vegas P, Stephan C, Castagna A, Antinori A, d'Arminio Monforte A, Torti C, Mussini C, Isern V, Calmy A, Teira R, Egger M, Grarup J, Chêne G, Collaboration of Observational HIV Epidemiological Research Europe (COHERE) in EuroCoord (2012). All-cause mortality in treated HIV-infected adults with CD4 ≥500/mm3 compared with the general population: evidence from a large European observational cohort collaboration. Int J Epidemiol.

[25] Thakur K, Boubour A, Saylor D, Das M, Bearden DR, Birbeck GL (2019). Global HIV neurology: a comprehensive review. AIDS.

[26] Olivier I, Cacabelos R, Naidoo V (2018). Risk factors and pathogenesis of HIV-associated neurocognitive disorder: the role of host genetics. Int J Mol Sci.

[27] Kumar S, Himanshu D, Tandon R, Atam V, Sawlani KK, Verma SK (2019). Prevalence of HIV associated neurocognitive disorder using Modified Mini Mental State Examination and its correlation with CD4 counts and anti-retroviral therapy. J Assoc Physicians India.

[28] Weber E, Blackstone K, Woods SP (2013). Cognitive neurorehabilitation of HIV-associated neurocognitive disorders: a qualitative review and call to action. Neuropsychol Rev.

[29] Nweke M, Nombeko M, Govender N, Akineplu AO (2021). Rehabilitation of HIV-associated neurocognitive disorder: a systematic scoping review of available interventions. Advances in Mental Health.

[30] Meintjes G, Moorhouse MA, Carmona S, Davies N, Dlamini S, van Vuuren C, Manzini T, Mathe M, Moosa Y, Nash J, Nel J, Pakade Y, Woods J, Van Zyl G, Conradie F, Venter F (2017). Adult antiretroviral therapy guidelines 2017. South Afr J HIV Med.

[31] Cui MY, Lin Y, Sheng JY, Zhang X, Cui RJ (2018). Exercise intervention associated with cognitive improvement in Alzheimer's disease. Neural Plast.

[32] McDermott A, Zaporojan L, McNamara P, Doherty CP, Redmond J, Forde C, Gormley J, Egaña M, Bergin C (2017). The effects of a 16-week aerobic exercise programme on cognitive function in people living with HIV. AIDS Care.

[33] Wykes T, Spaulding WD (2011). Thinking about the future cognitive remediation therapy--what works and could we do better?. Schizophr Bull.

[34] Boivin MJ, Busman RA, Parikh SM, Bangirana P, Page CF, Opoka RO, Giordani B (2010). A pilot study of the neuropsychological benefits of computerized cognitive rehabilitation in Ugandan children with HIV. Neuropsychology.

[35] Becker JT, Dew MA, Aizenstein HJ, Lopez OL, Morrow L, Saxton J, Tárraga L (2012). A pilot study of the effects of internet-based cognitive stimulation on neuropsychological function in HIV disease. Disabil Rehabil.

[36] Vance D, Cody S, Moneyham L (2017). Remediating HIV-associated neurocognitive disorders via cognitive training: a perspective on neurocognitive aging. Interdiscip Top Gerontol Geriatr.

[37] Chin LM, Keyser RE, Dsurney J, Chan L (2015). Improved cognitive performance following aerobic exercise training in people with traumatic brain injury. Arch Phys Med Rehabil.

[38] ten Brinke LF, Bolandzadeh N, Nagamatsu LS, Hsu CL, Davis JC, Miran-Khan K, Liu-Ambrose T (2015). Aerobic exercise increases hippocampal volume in older women with probable mild cognitive impairment: a 6-month randomised controlled trial. Br J Sports Med.

[39] Suzuki T, Shimada H, Makizako H, Doi T, Yoshida D, Ito K, Shimokata H, Washimi Y, Endo H, Kato T (2013). A randomized controlled trial of multicomponent exercise in older adults with mild cognitive impairment. PLoS One.

[40] O'Brien KK, Solomon P, Trentham B, MacLachlan D, MacDermid J, Tynan A, Baxter L, Casey A, Chegwidden W, Robinson G, Tran T, Wu J, Zack E (2014). Evidence-informed recommendations for rehabilitation with older adults living with HIV: a knowledge synthesis. BMJ Open.

[41] Dufour CA, Marquine MJ, Fazeli PL, Umlauf A, Henry BL, Zlatar Z, Montoya JL, Ellis RJ, Grant I, Moore DJ, HIV Neurobehavioral Research Program Group (2018). A longitudinal analysis of the impact of physical activity on neurocognitive functioning among HIV-infected adults. AIDS Behav.

[42] Monroe A, Zhang L, Jacobson L, Plankey M, Brown T, Miller E, Martin E, Becker J, Levine A, Ragin A, Sacktor N (2017). The association between physical activity and cognition in men with and without HIV infection. HIV Med.

[43] Nweke M, Mshunqane N, Govender N, Akinpelu O (2021). Physiological effects of physical activity on neurocognitive function in people living with HIV : a systematic review of intervention and observational studies. African Journal for Physical Activity and Health Sciences (AJPHES).

[44] Patten AR, Sickmann H, Hryciw BN, Kucharsky T, Parton R, Kernick A, Christie BR (2013). Long-term exercise is needed to enhance synaptic plasticity in the hippocampus. Learn Mem.

[45] Enette L, Vogel T, Fanon JL, Lang PO (2017). Effect of interval and continuous aerobic training on basal serum and plasma brain-derived neurotrophic factor values in seniors: a systematic review of intervention studies. Rejuvenation Res.

[46] Walker AJ, Bassett DR, Duey WJ, Howley ET, Bond V, Torok DJ, Mancuso P (1992). Cardiovascular and plasma catecholamine responses to exercise in blacks and whites. Hypertension.

[47] Ezema CI, Okwuchukwu CK, Amarachukwu CN, Nweke MC, Obiekwe C, Okafor CI, Okoye GC (2019). Effect of a single bout interval aerobic exercise on blood glucose level in type 2 diabetes mellitus patients. Ind. Jour. of Physioth. and Occupat. Therapy - An Inter. Jour.

[48] Lamina S, Okoye C (2011). Effect of interval training program on white blood cell count in the management of hypertension: A randomized controlled study. Niger Med J.

[49] American College of Sports Medicine (1993). Physical activity, physical fitness, and hypertension. Medicine & Science in Sports & Exercise.

[50] Carey C, Woods S, Rippeth J, Gonzalez R, Moore DJ, Marcotte TD, Grant IP, Heaton RK, HNRC Group (2004). Initial validation of a screening battery for the detection of HIV-associated cognitive impairment. Clin Neuropsychol.

[51] Towe S, Patel P, Meade CS (2017). The acceptability and potential utility of cognitive training to improve working memory in persons living with HIV: a preliminary randomized trial. J Assoc Nurses AIDS Care.

[52] Royal W, Cherner M, Carr J, Habib AG, Akomolafe A, Abimiku A, Charurat M, Farley J, Oluyemisi A, Mamadu I, Johnson J, Ellis R, McCutchan JA, Grant I, Blattner WA (2012). Clinical features and preliminary studies of virological correlates of neurocognitive impairment among HIV-infected individuals in Nigeria. J Neurovirol.

[53] Akolo C, Royal W, Cherner M, Okwuasaba K, Eyzaguirre L, Adebiyi R, Umlauf A, Hendrix T, Johnson J, Abimiku A, Blattner WA (2014). Neurocognitive impairment associated with predominantly early stage HIV infection in Abuja, Nigeria. J Neurovirol.

[54] Antinori A, Arendt G, Becker JT, Brew BJ, Byrd DA, Cherner M, Clifford DB, Cinque P, Epstein LG, Goodkin K, Gisslen M, Grant I, Heaton RK, Joseph J, Marder K, Marra CM, McArthur JC, Nunn M, Price RW, Pulliam L, Robertson KR, Sacktor N, Valcour V, Wojna VE (2007). Updated research nosology for HIV-associated neurocognitive disorders. Neurology.

[55] Beck AT (1961). An inventory for measuring depression. Arch Gen Psychiatry.

[56] Groth-Marnat G (1990). The handbook of psychological assessment, 2nd edition.

[57] Beck AT, Steer RA, Carbin MG (1988). Psychometric properties of the Beck Depression Inventory: Twenty-five years of evaluation. Clinical Psychology Review.

[58] Babor T, Higgins-Biddle J, Saunders JB, Monteiro MG (2001). AUDIT: the Alcohol Use Disorders Identification Test : guidelines for use in primary health care. World Health Organization.

[59] Skinner HA (1982). The drug abuse screening test. Addictive Behaviors.

[60] Sacktor N, Wong M, Nakasujja N, Skolasky R, Selnes O, Musisi S, Katabira E (2005). The International HIV Dementia Scale: A new rapid screening test for HIV dementia. AIDS.

[61] Benton AL, Hamsher KS, Sivan AB (1983). Multilingual aplasia examination, 2nd edition.

[62] Lezak MD, Howieson DB, Loring DW (2004). Neuropsychological assessment, 4th edition.

[63] Bizzozero I, Scotti S, Clerici F, Pomati S, Laiacona M, Capitani E (2013). On which abilities are category fluency and letter fluency grounded? A confirmatory factor analysis of 53 Alzheimer's dementia patients. Dement Geriatr Cogn Dis Extra.

[64] Brandt J, Benedict R (2001). Hopkins Verbal Learning Test-Revised Professional Manual.

[65] Lacritz LH, Cullum CM, Weiner MF, Rosenberg RN (2010). Comparison of the Hopkins Verbal Learning Test-Revised to the California Verbal Learning Test in Alzheimer's disease. Applied Neuropsychology.

[66] Reitan RM, Wolfson D (1993). The Halstead-Reitan neuropsychological test battery: Theory and clinical interpretation.

[67] Llinàs-Reglà J, Vilalta-Franch J, López-Pousa S, Calvó-Perxas L, Torrents Rodas D, Garre-Olmo J (2017). The Trail Making Test. Assessment.

[68] Stern R, White T (2009). NAB Digits Forward/Digits Backward Test: Professional Manual.

[69] Hsiung P, Fang C, Chang Y, Chen M, Wang J (2005). Comparison of WHOQOL-bREF and SF-36 in patients with HIV infection. Qual Life Res.

[70] Tumusiime D, Stewart A, Venter F (2015). Effect of physiotherapeutic exercises on peripheral neuropathy, functional limitations of lower extremity and quality of life in people with HIV. Physiotherapy.

[71] Jang Y, Hsieh C, Wang Y, Wu Y (2004). A validity study of the WHOQOL-BREF assessment in persons with traumatic spinal cord injury. Arch Phys Med Rehabil.

[72] International Classification of Functioning, Disability and Health (ICF). World Health Organization.

[73] Morley D, Dummett S, Kelly L, Dawson J, Fitzpatrick R, Jenkinson C (2016). Validation of the Oxford Participation and Activities Questionnaire. PROM.

[74] Kelly L, Dummett S, Dawson J, Churchman D, Fitzpatrick R, Jenkinson C, Morley D (2016). Pretesting an e-based version of the Oxford Participation & Activities Questionnaire (Ox-Paq). Value in Health.

[75] Herdman M, Gudex C, Lloyd A, Janssen M, Kind P, Parkin D, Bonsel G, Badia X (2011). Development and preliminary testing of the new five-level version of EQ-5D (EQ-5D-5L). Qual Life Res.

[76] Warburton DE, Jamnik VK, Bredin SS, McKenzie DC, Stone J, Shephard RJ, Gledhill N (2011). Evidence-based risk assessment and recommendations for physical activity clearance: an introduction. Appl Physiol Nutr Metab.

[77] Whitfield GP, Pettee Gabriel KK, Rahbar MH, Kohl HW (2014). Application of the American Heart Association/American College of Sports Medicine Adult preparticipation screening checklist to a nationally representative sample of US adults aged ≥40 years from the National Health and Nutrition Examination Survey 2001 to 2004. Circulation.

[78] Musa DI, Ibrahim DM, Toriola AL (2002). Cardiorespiratory fitness and risk factors of CHD in pre-adolescent Nigerian girls. Journal of Human Movement Studies.

[79] MacDougall JD, Wenger HA, Wenger HA, Green HJ, MacDougall JD (1991). Physiological testing of the high performance athletes.

[80] (2001). International standards for anthropometric assessment.

[81] Barbieri M (2003). Is chronic inflammation a determinant of blood pressure in the elderly?. American Journal of Hypertension.

[82] Campbell PJ, Aurelius S, Blowes G, Harvey D (1997). Decrease in CD4 lymphocyte counts with rest; implications for the monitoring of HIV infection. Int J STD AIDS.

[83] Nnadi O, Liwenga ET, Lyimo JG, Madukwe MC (2019). Impacts of variability and change in rainfall on gender of farmers in Anambra, Southeast Nigeria. Heliyon.

[84] Antiretroviral therapy for HIV infection in adults and adolescents: recommendations for a public health approach. World Health Organization.

[85] (2010). Report on the Global HIV/AIDS Epidemic. UNAIDS.

[86] Hardie D, Korsman S, Ameer S, Vojnov L, Hsiao NY (2019). Reliability of plasma HIV viral load testing beyond 24 hours: Insights gained from a study in a routine diagnostic laboratory. PLoS One.

[87] Stark NJ (1999). Managing Adverse Events and Effects during Clinical Trials. Medical Device & Diagnostic Industry.

[88] Niemeijer A, Lund H, Stafne SN, Ipsen T, Goldschmidt C, Jørgensen CT, Juhl CB (2020). Adverse events of exercise therapy in randomised controlled trials: a systematic review and meta-analysis. Br J Sports Med.

[89] Bernstein SL, Feldman J (2015). Incentives to participate in clinical trials: practical and ethical considerations. Am J Emerg Med.

[90] Mapstone J, Elbourne D, Roberts I (2007). Strategies to improve recruitment to research studies. Cochrane Database Syst Rev.

[91] Kavanagh J, Trouton A, Oakley A, Powell C (2006). A systematic review of the evidence for incentive schemes to encourage positive health and other social behaviours in young people. EPPI Report.

[92] Adams J, Giles EL, Robalino S, McColl E, Sniehotta FF (2012). A systematic review of the use of financial incentives and penalties to encourage uptake of healthy behaviors: protocol. Syst Rev.

