# Correction: Aerobic Exercise in HIV-Associated Neurocognitive Disorders: Protocol for a Randomized Controlled Trial

**DOI:** 10.2196/40486

**Published:** 2022-06-28

**Authors:** Martins Nweke, Nombeko Mshunqane, Nalini Govender, Aderonke Akinpelu, Adesola Ogunniyi

**Affiliations:** 1 Department of Physiotherapy Faculty of Health Sciences University of Pretoria Enugu Nigeria; 2 Department of Physiotherapy Faculty of Health Sciences University of Pretoria Pretoria South Africa; 3 Department of Basic Medical Sciences Faculty of Health Sciences Durban University of Technology Durban South Africa; 4 Department of Physiotherapy Faculty of Clinical Sciences University of Ibadan Ibadan Nigeria; 5 Department of Medicine Faculty of Clinical Sciences University of Ibadan Ibadan Nigeria

In “Aerobic Exercise in HIV-Associated Neurocognitive Disorders: Protocol for a Randomized Controlled Trial” (JMIR Res Protoc 2022;11(1):e29230) the authors noted one error.

The name of the second author was incorrectly displayed as:

Mshunqane Nombeko

It has now been corrected to:

Nombeko Mshunqane

The correction will appear in the online version of the paper on the JMIR Publications website on June 28, 2022, together with the publication of this correction notice. Because this was made after submission to PubMed, PubMed Central, and other full-text repositories, the corrected article has also been resubmitted to those repositories.

