# Supplementary material for: Aerobic Exercise in HIV-Associated Neurocognitive Disorders: Protocol for a Randomized Controlled Trial
Source: JMIR Res Protoc. 2022 Jan 31;11(1):e29230. doi: 10.2196/29230 (PMC8844984; doi:10.2196/29230)
Supplement: Multimedia Appendix 2 [file resprot_v11i1e29230_app2.pdf]

SUPPL. FILE II: ALCOHOL USE DISORDER IDENTIFICATION TEST (AUDIT)

The Alcohol Use Disorders Identification Test: Interview Version

Read questions as written. Record answers carefully. Begin the AUDIT by saying "Now I am going to ask you some questions about your use of alcoholic beverages during this past year." Explain what is meant by "alcoholic beverages" by using local examples of beer, wine, vodka, etc. Code answers in terms of "standard drinks". Place the correct answer number in the box at the right.

1. How often do you have a drink containing alcohol?
   (0) Never [Skip to Qs 9-10]
   (1) Monthly or less
   (2) 2 to 4 times a month
   (3) 2 to 3 times a week
   (4) 4 or more times a week

2. How many drinks containing alcohol do you have on a typical day when you are drinking?
   (0) 1 or 2
   (1) 3 or 4
   (2) 5 or 6
   (3) 7, 8, or 9
   (4) 10 or more

3. How often do you have six or more drinks on one occasion?
   (0) Never
   (1) Less than monthly
   (2) Monthly
   (3) Weekly
   (4) Daily or almost daily
   Skip to Questions 9 and 10 if Total Score for Questions 2 and 3 = 0

4. How often during the last year have you found that you were not able to stop drinking once you had started?
   (0) Never
   (1) Less than monthly
   (2) Monthly
   (3) Weekly
   (4) Daily or almost daily

5. How often during the last year have you failed to do what was normally expected from you because of drinking?
   (0) Never
   (1) Less than monthly
   (2) Monthly
   (3) Weekly
   (4) Daily or almost daily

6. How often during the last year have you needed a first drink in the morning to get yourself going after a heavy drinking session?
   (0) Never
   (1) Less than monthly
   (2) Monthly
   (3) Weekly
   (4) Daily or almost daily

7. How often during the last year have you had a feeling of guilt or remorse after drinking?
   (0) Never
   (1) Less than monthly
   (2) Monthly
   (3) Weekly
   (4) Daily or almost daily

8. How often during the last year have you been unable to remember what happened the night before because you had been drinking?
   (0) Never
   (1) Less than monthly
   (2) Monthly
   (3) Weekly
   (4) Daily or almost daily

9. Have you or someone else been injured as a result of your drinking?
   (0) No
   (2) Yes, but not in the last year
   (4) Yes, during the last year

10. Has a relative or friend or a doctor or another health worker been concerned about your drinking or suggested you cut down?
   (0) No
   (2) Yes, but not in the last year
   (4) Yes, during the last year

Record total of specific items here

If total is greater than recommended cut-off, consult User's Manual.
