# Supplementary material for: Aerobic Exercise in HIV-Associated Neurocognitive Disorders: Protocol for a Randomized Controlled Trial
Source: JMIR Res Protoc. 2022 Jan 31;11(1):e29230. doi: 10.2196/29230 (PMC8844984; doi:10.2196/29230)
Supplement: Multimedia Appendix 3 [file resprot_v11i1e29230_app3.pdf]

SUPL FILE III: DRUG ABUSE SCREENING TEST (DAST)

General Instructions

"Drug use" refers to (1) the use of prescribed or over-the-counter drugs in excess of the directions, and (2) any non-medical use of drugs.

The various classes of drugs may include cannabis (marijuana, hashish), solvents (e.g., paints thinner), tranquilizer (e.g., Valium), barbiturates, cocaine, stimulants (e.g., speed), hallucinogens (e.g., LSD) or narcotics (e.g., heroin). The questions do not include alcoholic beverages. Please answer every question. If you have difficulty with a statement, then chose the response that is mostly right.

These questions refer to drug use in the past 12 months. Please answer No or Yes.

1. Have you used drugs other than those required for medical reasons?

   ☐ No ☐ Yes

2. Do you use more than one drug at a time?

   ☐ No ☐ Yes

3. Are you always able to stop using drugs when you want to?

   ☐ No ☐ Yes

4. Have you had "blackouts" or "flashbacks" as a result of drug use?

   ☐ No ☐ Yes

5. Do you ever feel bad or guilty about drug use?

   ☐ No ☐ Yes

6. Does your spouse (or parents) ever complain about your involvement with drugs?

   ☐ No ☐ Yes

7. Have you neglected your family because of your use of drugs?

   ☐ No ☐ Yes

8. Have you engaged in illegal activities in order to obtain drugs?

   ☐ No ☐ Yes

9. Have you ever experienced withdrawal symptoms (felt sick) when you stopped taking drugs?

   ☐ No ☐ Yes

10. Have you had medical problems as a result of a result of your drug use (e.g., memory loss, hepatitis, convulsion, bleeding, etc.)? No ☐ Yes ☐

    Score:
