# Supplementary material for: Aerobic Exercise in HIV-Associated Neurocognitive Disorders: Protocol for a Randomized Controlled Trial
Source: JMIR Res Protoc. 2022 Jan 31;11(1):e29230. doi: 10.2196/29230 (PMC8844984; doi:10.2196/29230)
Supplement: Multimedia Appendix 4 [file resprot_v11i1e29230_app4.pdf]

Mental Disorders Screening
Dementia: IHDS
Anxiety: GAD-2
Anxiety: GAD-7
Depression: PHQ-2
Depression: PHQ-9
PTSD: PC-PTSD-5

Substance Use Screening
Alcohol: AUDIT-C
Alcohol: CAGE
CAGE-AID
Drug Abuse: DAST-10
Drug Abuse: TICS
Opioid: Risk Tool

Clinical Calculators
APRI Calculator
BMI Calculator
CrCl Calculator
CTP Calculator
FIB-4 Calculator
FEPO4 Calculator
GFR Calculator

International HIV Dementia Scale (IHDS)

Share

Here is a short summary about the tool explaining in brief its efficacy.

- This tool is not meant to be viewed by the patient during the test. The provider should use it as a guide and demonstrate all actions to the patient with their own hands.
- The timers will emit audio cues at the beginning and end of the time period.
- To perform this test follow the steps in order below:

Memory-Registration

dog **hat** **bean** **red**

1. Give four words to recall (**dog, hat, bean, red**) – 1 second to say each.
2. Then ask the patient all four words after you have said them,
3. Repeat words if the patient does not recall them all immediately.
4. Tell the patient you will ask for recall of the words again a bit later.

1. Motor Speed

0-4 points

Have the patient tap the first two fingers of the non-dominant hand as widely and as quickly as possible.

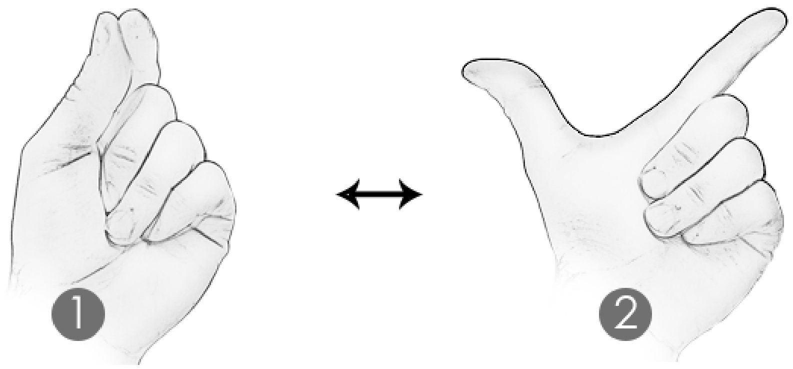

Count the number of taps performed in 5 seconds:

5 seconds

Start Timer

Record Number of Taps:

0 or more

Scoring:
≥ 15 taps = **4 points**
11-14 taps = **3 points**
7-10 taps = **2 points**
3-6 taps = **1 point**
0-2 taps = **0 points**

2. Psychomotor Speed 0-4 points

Have the patient perform the following sequence of movements with the non-dominant hand as quickly as possible:

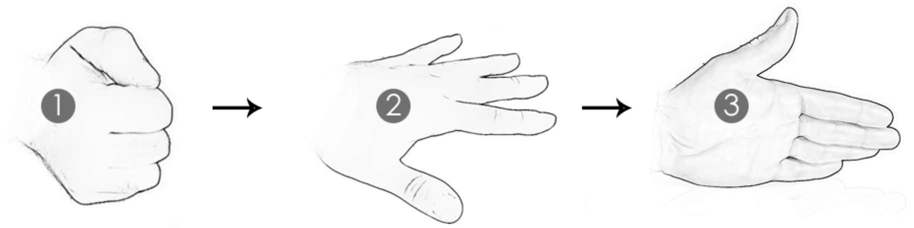

1. Clench hand in fist on flat surface.
2. Put hand flat on surface with palm down.
3. Put hand perpendicular to flat surface on the side of the 5th digit.

Demonstrate and have patient perform twice for practice.

Count the number of sequences performed in 10 seconds:

10 seconds

Start Timer

Record Number of Sequences:

0 or more

Scoring:
≥ 4 sequences = **4 points**
3 sequences = **3 points**
2 sequences = **2 points**
1 sequence = **1 point**
unable to perform = **0 points**

3. Memory Recall 0-4 points

Ask the patient to recall the four words:

Select the words recalled below: (1 point each)

dog hat bean red

For words not recalled, prompt with a semantic clue as follows:

Select the words recalled using clue. (0.5 points each)

animal? dog

piece of clothing? hat

vegetable? bean

color? red

Results and Interpretation

Final Score: **0 points**

Interpretation:

- This is the sum of the scores on items 1-3. The maximum possible score is 12 points. A patient with a score of ≤10 should be evaluated further for possible dementia.

Acknowledgement

- The International HIV Dementia Scale (IHDS) was developed by Dr. Ned C. Sacktor, Professor of Neurology, Johns Hopkins Medicine.
- Reproduced with permission from Dr. Ned C. Sacktor.

Sources

- Sacktor NC, Wong M, Nakasujja N, et al. The International HIV Dementia Scale: a new rapid screening test for HIV dementia. AIDS. 2005;19:1367-74

This calculator operates entirely from your device.
No input variables or data is transmitted between your computer and our servers.

Funded by a grant from the
Health Resources and Services Administration (HRSA)

CME provided by

HIV Medications

Single-Tablet Regimens
Entry Inhibitors
Nucleoside Reverse Transcriptase Inhibitors
Non-Nucleoside Reverse Transcriptase Inhibitors
Integrase Inhibitors
Protease Inhibitors
Pharmacokinetic Enhancers

Course Modules

Screening and Diagnosis
Basic HIV Primary Care
Antiretroviral Therapy
Co-Occurring Conditions
Prevention of HIV
Key Populations

Question Bank
Clinical Challenges
Master Bibliography

Contributors
Content Bundles

Tools & Calculators

Mental Disorders Screening
Substance Use Screening
Clinical Calculators

Copyright © 2019 National HIV Curriculum
Version 2.7.2-c - AWS

Contact Us | Terms and Conditions | Privacy Policy
CE (CNE/CME) Notices | Copyright and Attribution Notices

This project is supported by the Health Resources and Services Administration (HRSA) of the U.S. Department of Health and Human Services (HHS) under grant number U10HA32104. No percentage of this project was financed with non-governmental sources. This information or content and conclusions are those of the authors and should not be construed as the official position or policy of, nor should any endorsements by inferred by HRSA, HHS or the U.S. Government.

part of the IDEA platform
