# Supplementary material for: Aerobic Exercise in HIV-Associated Neurocognitive Disorders: Protocol for a Randomized Controlled Trial
Source: JMIR Res Protoc. 2022 Jan 31;11(1):e29230. doi: 10.2196/29230 (PMC8844984; doi:10.2196/29230)
Supplement: Multimedia Appendix 5 [file resprot_v11i1e29230_app5.docx]

**VERBAL FLUENCY TEST**

In this test, I want you to say as many words as you can within 3minutes; 1 minute for each letter. There are three letters F, A, S. Now, I will show you how to do it using letter “C”: cable, car, coffee, etc. Did you get that? Please avoid using name of person, animal or place. Are you ready for the test now? Remember you will start with “F” and mention all the words you can remember that starts with “F” within 1minute; at the end you will do the same with “A” and the “S”.

Start- set the timer for 1minutes (60 seconds)

F:

Start- set the timer for 1minutes (60 seconds)

A:

Start- set the timer for 1minutes (60 seconds)

S:
