# Supplementary material for: Aerobic Exercise in HIV-Associated Neurocognitive Disorders: Protocol for a Randomized Controlled Trial
Source: JMIR Res Protoc. 2022 Jan 31;11(1):e29230. doi: 10.2196/29230 (PMC8844984; doi:10.2196/29230)
Supplement: Multimedia Appendix 6 [file resprot_v11i1e29230_app6.pdf]

THE HOPKINS VERBAL LEARNING TEST

Abstract

A new test of verbal learning and memory, the Hopkins Verbal Learning Test, was developed. The test consists of three trials of free-recall of a 12-item, semantically categorized list, followed by yes/no recognition. Six parallel forms yielded equivalent results in normals. The performance of patients with Alzheimer's disease and chronic amnesia is described. The test is likely to be useful in patients too impaired for more comprehensive memory assessments and where repeated testing is necessary.

Clinical practice and research in neuro-psychology often require brief, repeated assessments of the same patient over time. Most of the newer clinical memory tests are of limited utility for this purpose because of their length, complexity and/or lack of parallel forms. The WMS-R, for example, require 45 to 60 minutes for administration and, at present, is available in only one form. The California Learning Test (Delis, Kramer, Kaplan & Ober, 1986) is gaining popularity as a relatively comprehensive verbal memory test, and an alternate form has been developed (Delis, et al), but its length and complexity often make it unwieldy for use with demented or otherwise difficult-to-test patients.

Description of the Test

Each form of the Hopkins Verbal Learning Test (HVLT) consists of a 12-item word list, composed of four words from each of the three semantic categories. The subject is instructed to listen carefully as the examiner reads the word list and attempt to memorize the words. The word list is then read to the subject at the approximate rate of one word every 2 seconds. The patient's free recall of the list is recorded. The same procedure is repeated for two more trials. After the third learning trial, the patient is read 24 words and is asked to say "yes" after each word that appeared on the recall list (12 targets) and "no" after each word that did not (12 distractors). Half of the distractors are drawn from the same semantic categories as the targets (related distractors) and half are drawn from other categories (unrelated distractors).

There are several advantages of the HVLT over many existing memory tests. First, it requires no more than 10 minutes to administer. Second, it is well-tolerated by even moderately to severely demented patients, while not having a ceiling effect (in recall) in neurologically normal subjects. Third, the existence of six comparable forms makes the HVLT particularly useful in research where patients are assessed at frequent intervals.

The HVLT was recently employed in a study of the effects of intravenous physostigmine in Alzheimer's disease (Tune et al, in press). Patients received an intravenous infusion of one of three doses of drug, or placebo, on four consecutive days. Memory and other cognitive functions had to be administered rapidly, during the 20 minutes of maximum drug effect. The HVLT proved an ideal test for this purpose.

HOPKINS VERBAL LEARNING TEST
Form 1: four-legged animals, precious stones, human dwellings

Part A: Free Recall

	Trial 1	Trial 2	Trial 3
EMERALD	________	________	________
HORSE	________	________	________
TENT	________	________	________
SAPPHIRE	________	________	________
HOTEL	________	________	________
CAVE	________	________	________
OPAL	________	________	________
TIGER	________	________	________
PEARL	________	________	________
COW	________	________	________
HUT	________	________	________
# CORRECT	________	________	________

HORSE	ruby*	CAVE	balloon	coffee	LION
house*	OPAL	TIGER	boat	scarf	PEARL
HUT	EMERALD	SAPPHIRE	dog*	apartment*	penny
TENT	mountain	cat*	HOTEL	COW	diamond*

Part B: Recognition:

# True-Positives:______________/12

# False-Positive Errors: Related:____________/6 Unrelated:___________/6

Discrimination Index: (# True-Positives) – (# False-Positives) = ________________

HOPKINS VERBAL LEARNING TEST
Form 2: kitchen utensils, alcoholic beverages, weapons

Part A: Free Recall

	Trial 1	Trial 2	Trial 3
FORK	________	________	________
RUM	________	________	________
PAN	________	________	________
PISTOL	________	________	________
SWORD	________	________	________
SPATULA	________	________	________
BOURBON	________	________	________
VODKA	________	________	________
POT	________	________	________
COW	________	________	________
HUT	________	________	________
WINE	________	________	________
# CORRECT	________	________	________

Part B: Recognition:

spoon*	PISTOL	doll	whiskey*	FORK	POT
harmonica	can opener*	SWORD	pencil	gun*	VODKA
knife*	RUM	trout	BOMB	PAN	gold
WINE	lemon	SPATULA	BOURBON	beer*	RIFLE

# True-Positives:______________/12

# False-Positive Errors: Related:____________/6 Unrelated:___________/6

Discrimination Index: (# True-Positives) – (# False-Positives) = ________________

HOPKINS VERBAL LEARNING TEST
Form 3: musical instruments, fuels, food flavorings

Part A: Free Recall

	Trial 1	Trial 2	Trial 3
SUGAR	________	________	________
TRUMPET	________	________	________
VIOLIN	________	________	________
COAL	________	________	________
GARLIC	________	________	________
KEROSINE	________	________	________
VANILLA	________	________	________
WOOD	________	________	________
CLARINET	________	________	________
FLUTE	________	________	________
CINNAMON	________	________	________
GASOLINE	________	________	________
# CORRECT	________	________	________

Part B: Recognition:

pepper*	GARLIC	WOOD	drum*	oil*	SUGAR
Harmonica	salt*	priest	chair	COAL	CLARINET
TRUMPET	basement	CINNAMON	FLUTE	electricity*	Moon
KEROSINE	VANILLA	GASOLINE	sand	piano*	VIOLIN

# True-Positives:______________/12

# False-Positive Errors: Related:____________/6 Unrelated:___________/6

Discrimination Index: (# True-Positives) – (# False-Positives) = ________________

HOPKINS VERBAL LEARNING TEST
Form 4: birds, articles of clothing, carpenter's tools

Part A: Free Recall

	Trial 1	Trial 2	Trial 3
CANARY	________	________	________
SHOES	________	________	________
EAGLE	________	________	________
BLOUSE	________	________	________
NAILS	________	________	________
CROW	________	________	________
BLUEBIRD	________	________	________
SCREWDRIVER	________	________	________
PANTS	________	________	________
CHISEL	________	________	________
SKIRT	________	________	________
WRENCH	________	________	________
# CORRECT	________	________	________

Part B: Recognition:

BLUEBIRD	shirt*	CHISEL	EAGLE	chocolate	robin*
chapel	SCREWDRIVER	CROW	sparrow*	WRENCH	PANTS
NAILS	socks*	child	SHOES	hair	hammer*
CANARY	apple	SKIRT	saw*	silver	BLOUSE

True-Positives:______________/12

False-Positive Errors: Related:____________/6 Unrelated:___________/6

Discrimination Index: (# True-Positives) – (# False-Positives) = ________________

HOPKINS VERBAL LEARNING TEST
Form 5: occupations/professions, sports, vegetables

Part A: Free Recall

	Trial 1	Trial 2	Trial 3
TEACHER	________	________	________
BASKETBALL	________	________	________
LETTUCE	________	________	________
DENTIST	________	________	________
TENNIS	________	________	________
BEAN	________	________	________
ENGINEER	________	________	________
POTATO	________	________	________
PROFESSOR	________	________	________
GOLF	________	________	________
CORN	________	________	________
SOCCER	________	________	________
# CORRECT	________	________	________

Part B: Recognition:

# True-Positives:______________/12

TENNIS	football*	PROFESSOR	spinach*	lawyer*	submarine
GOLF	DENTIST	LETTUCE	spider	water	BEAN
BASKETBALL	doctor*	CORN	baseball*	TEACHER	snake
carrot*	ENGINEER	glove	SOCCER	POTATO	tulip

# False-Positive Errors: Related:____________/6 Unrelated:___________/6

Discrimination Index: (# True-Positives) – (# False-Positives) = ________________

HOPKINS VERBAL LEARNING TEST
Form 6: fish, parts of a building, phenomens

Part A: Free Recall

	Trial 1	Trial 2	Trial 3
SHARK	________	________	________
WALL	________	________	________
HERRING	________	________	________
RAIN	________	________	________
FLOOR	________	________	________
HAIL	________	________	________
CATFISH	________	________	________
ROOF	________	________	________
SALMON	________	________	________
STORM	________	________	________
CEILING	________	________	________
SNOW	________	________	________
# CORRECT	________	________	________

Part B: Recognition:

# True-Positives:______________/12

HAIL	bass*	SNOW	bank	FLOOR	mustard
window*	CEILING	canyon	RAIN	ladder	STORM
HERRING	SALMON	tornado*	trout*	melon	ROOF
SHARK	hurricane*	elbow	CATFISH	WALL	door*

# False-Positive Errors: Related:____________/6 Unrelated:___________/6

Discrimination Index: (# True-Positives) – (# False-Positives) = ________________
