# Supplementary material for: Aerobic Exercise in HIV-Associated Neurocognitive Disorders: Protocol for a Randomized Controlled Trial
Source: JMIR Res Protoc. 2022 Jan 31;11(1):e29230. doi: 10.2196/29230 (PMC8844984; doi:10.2196/29230)

Trail Making Test (TMT) Parts A & B

Instructions:

Both parts of the Trail Making Test consist of 25 circles distributed over a sheet of paper. In Part A, the circles are numbered 1 – 25, and the patient should draw lines to connect the numbers in ascending order. In Part B, the circles include both numbers (1 – 13) and letters (A – L); as in Part A, the patient draws lines to connect the circles in an ascending pattern, but with the added task of alternating between the numbers and letters (i.e., 1-A-2-B-3-C, etc.). The patient should be instructed to connect the circles as quickly as possible, without lifting the pen or pencil from the paper. Time the patient as he or she connects the "trail." If the patient makes an error, point it out immediately and allow the patient to correct it. Errors affect the patient's score only in that the correction of errors is included in the completion time for the task. It is unnecessary to continue the test if the patient has not completed both parts after five minutes have elapsed.

Step 1: Give the patient a copy of the Trail Making Test Part A worksheet and a pen or pencil.

Step 2: Demonstrate the test to the patient using the sample sheet (Trail Making Part A – *SAMPLE*).

Step 3: Time the patient as he or she follows the “trail” made by the numbers on the test.

Step 4: Record the time.

Step 5: Repeat the procedure for Trail Making Test Part B.

Scoring:

Results for both TMT A and B are reported as the number of seconds required to complete the task; therefore, higher scores reveal greater impairment.

	**Average**	**Deficient**	**Rule of Thumb**
**Trail A**	29 seconds	> 78 seconds	Most in 90 seconds
**Trail B**	75 seconds	> 273 seconds	Most in 3 minutes

Sources:

- Corrigan JD, Hinkeldey MS. Relationships between parts A and B of the Trail Making Test. *J Clin Psychol*. 1987;43(4):402–409.
- Gaudino EA, Geisler MW, Squires NK. Construct validity in the Trail Making Test: what makes Part B harder? *J Clin Exp Neuropsychol*. 1995;17(4):529-535.
- Lezak MD, Howieson DB, Loring DW. *Neuropsychological Assessment*. 4th ed. New York: Oxford University Press; 2004.
- Reitan RM. Validity of the Trail Making test as an indicator of organic brain damage. *Percept Mot Skills*. 1958;8:271-276.

Trail Making Test Part A

Patient's Name: ________________________________ Date: ______________________

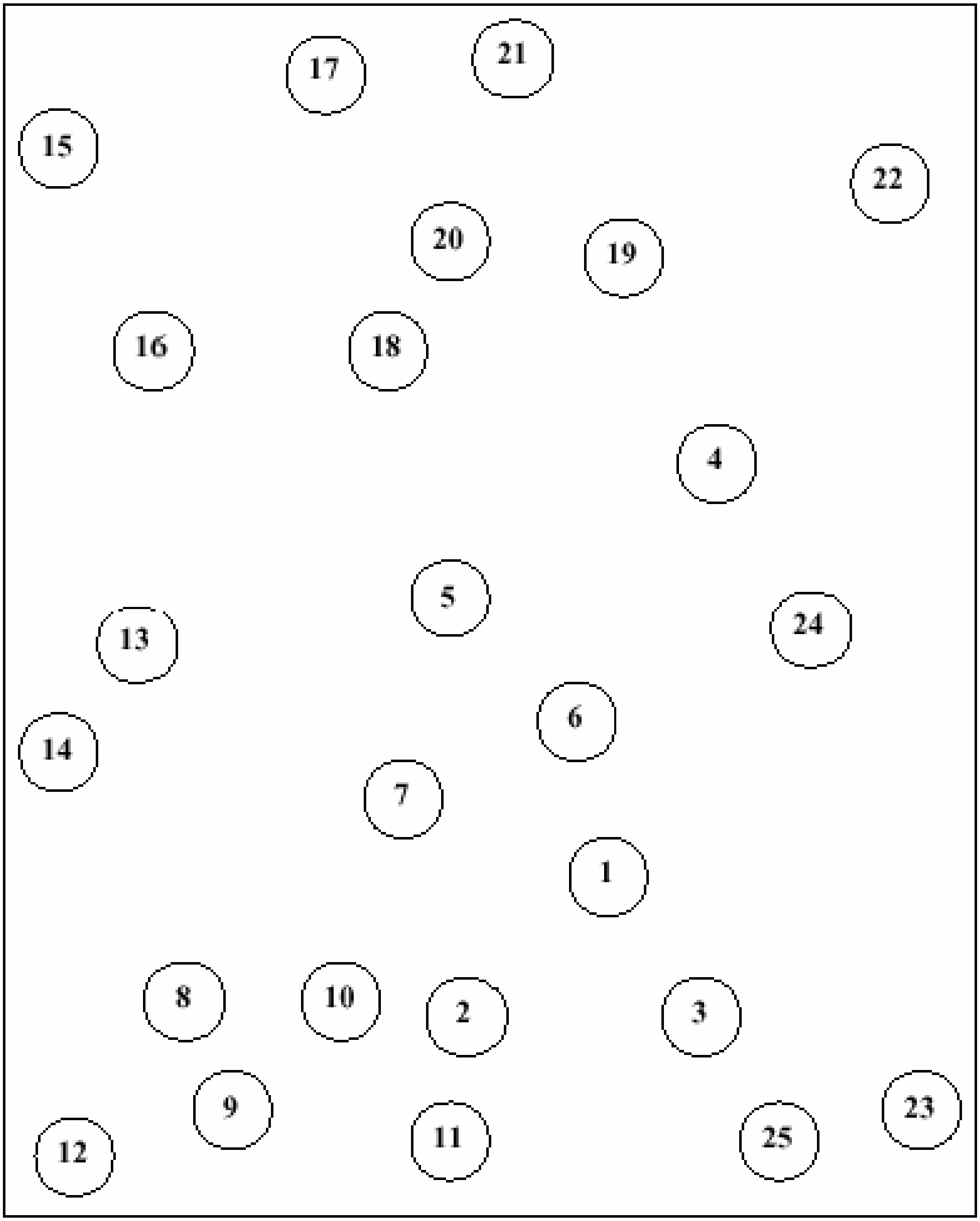

Trail Making Test Part A – *SAMPLE*

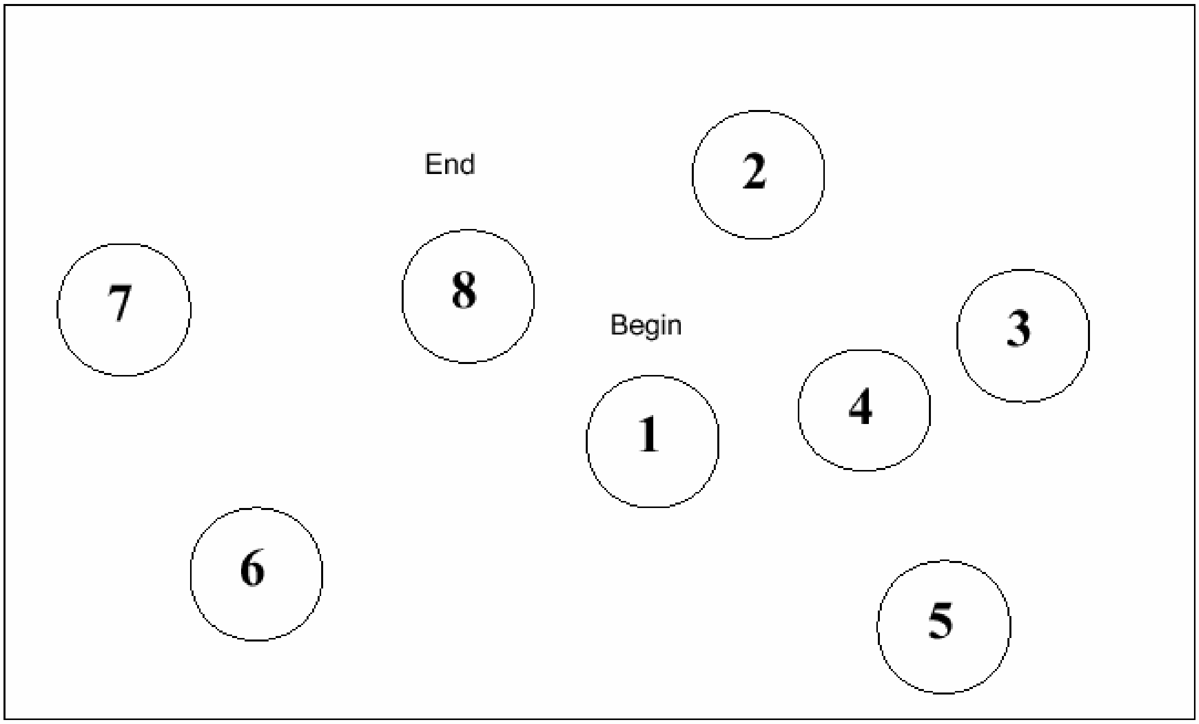

Trail Making Test Part B

Patient's Name: ________________________________ Date: ______________________

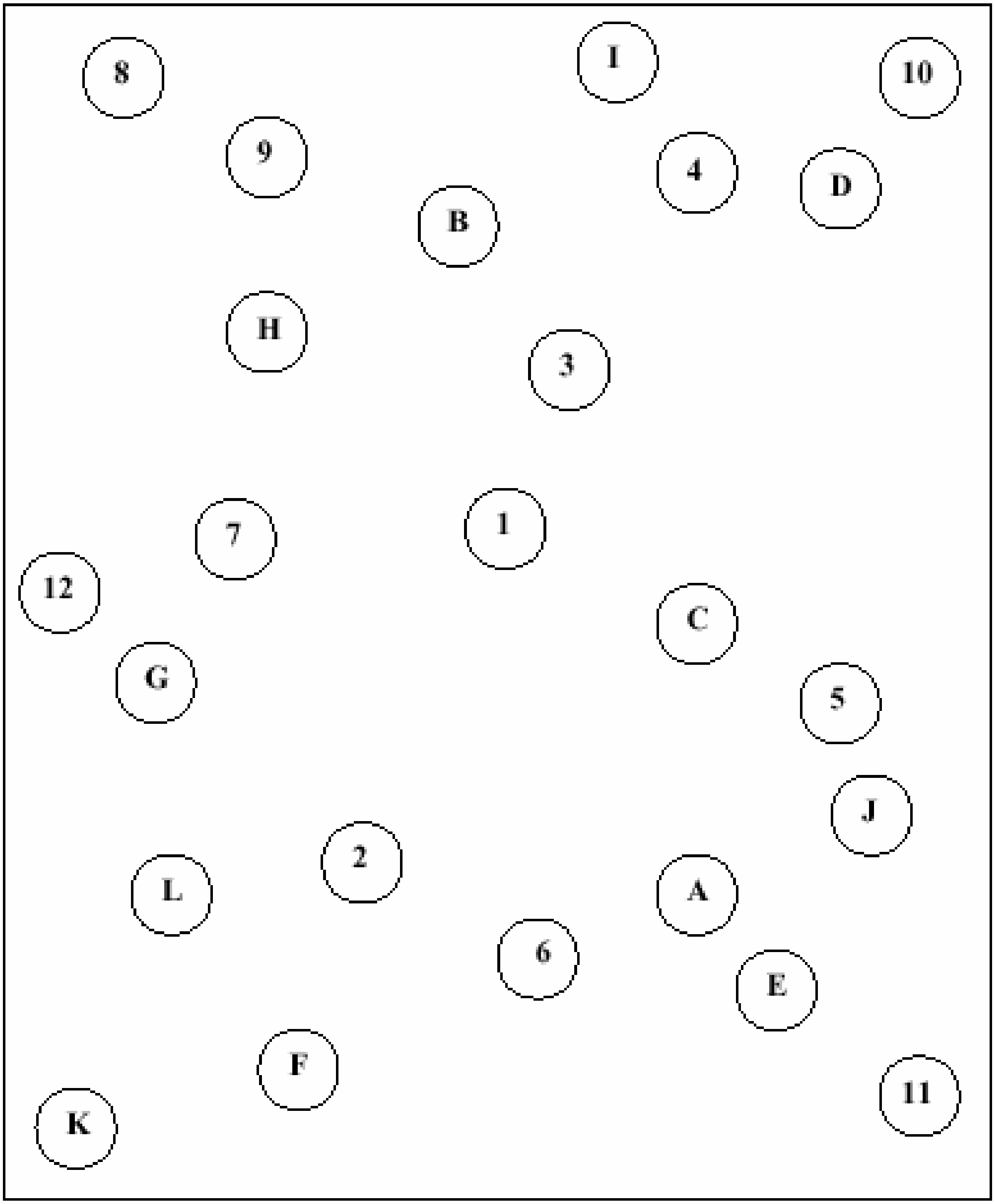

Trail Making Test Part B – *SAMPLE*

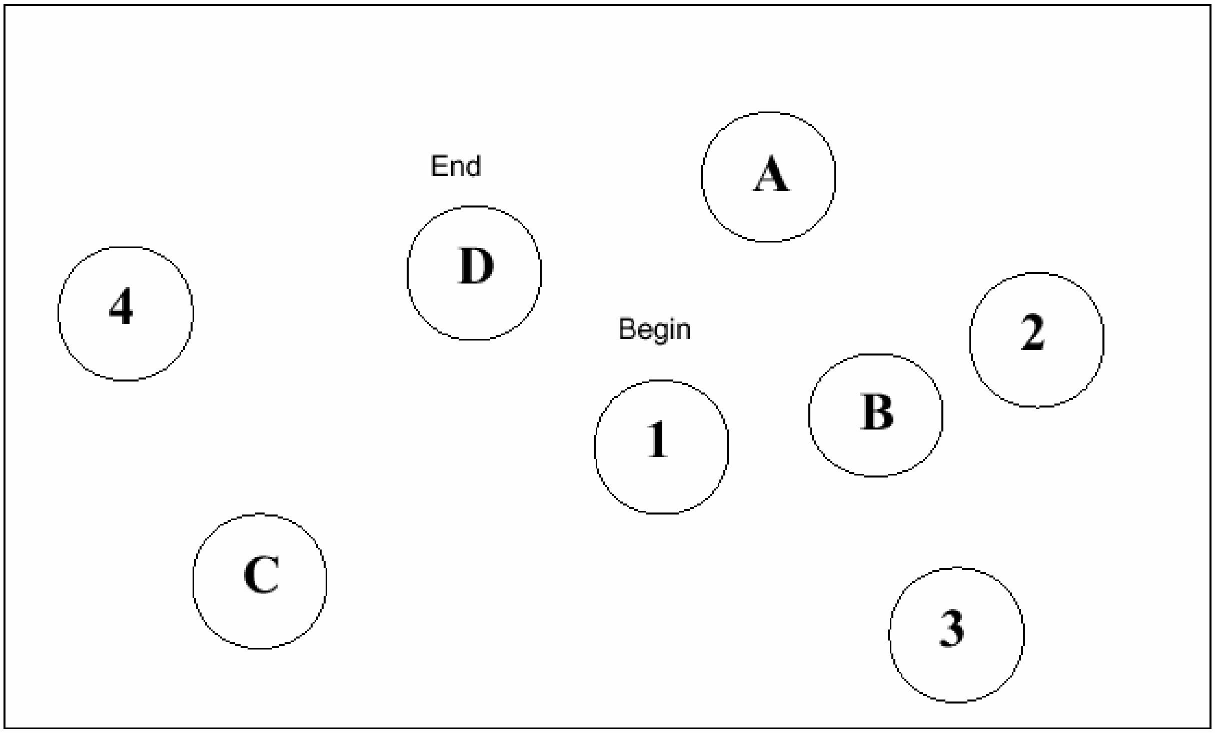

Supplement: Multimedia Appendix 7 [file resprot_v11i1e29230_app7.pdf]
