# Supplementary figures and images for: Aerobic Exercise in HIV-Associated Neurocognitive Disorders: Protocol for a Randomized Controlled Trial
Source: JMIR Res Protoc. 2022 Jan 31;11(1):e29230. doi: 10.2196/29230 (PMC8844984; doi:10.2196/29230)

**5** DIGIT SPAN TESTS (FORWARD AND BACKWARD)


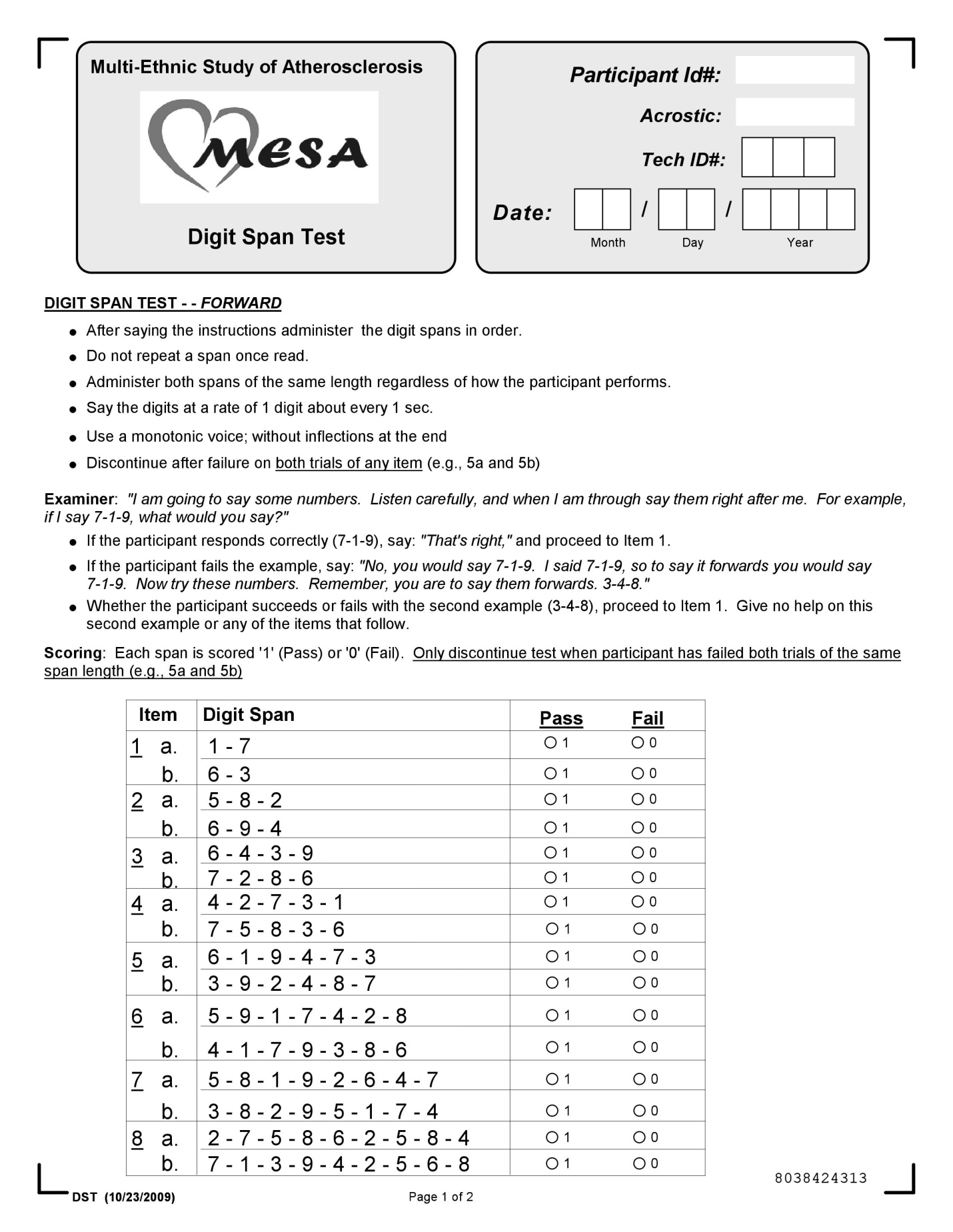


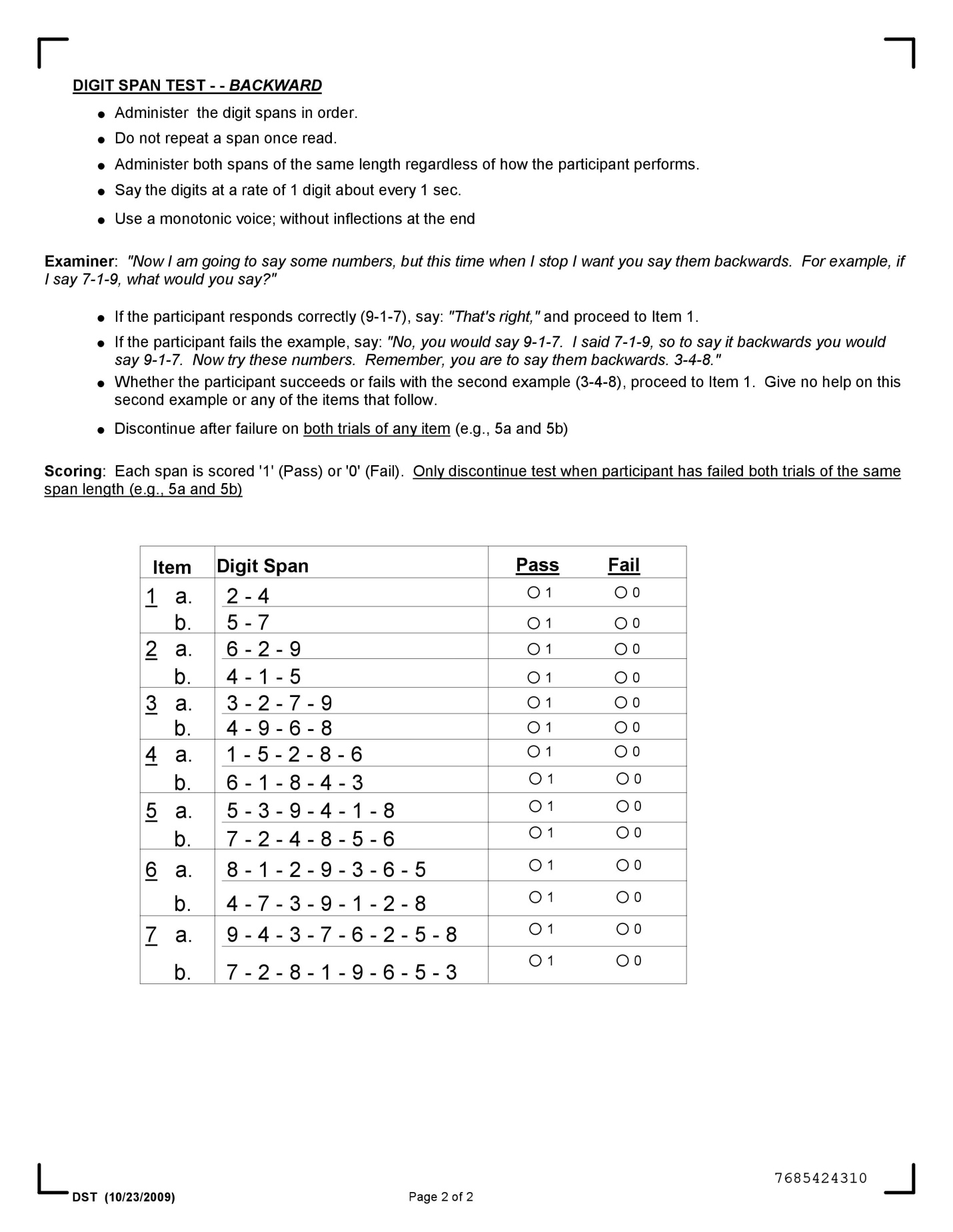

Supplement: Multimedia Appendix 8 [file resprot_v11i1e29230_app8.docx]
