# Supplementary material for: Aerobic Exercise in HIV-Associated Neurocognitive Disorders: Protocol for a Randomized Controlled Trial
Source: JMIR Res Protoc. 2022 Jan 31;11(1):e29230. doi: 10.2196/29230 (PMC8844984; doi:10.2196/29230)
Supplement: Multimedia Appendix 9 [file resprot_v11i1e29230_app9.pdf]

general assessment series

try this:®

Best Practices in Nursing Care to Older Adults

From The Hartford Institute for Geriatric Nursing, New York University Rory Meyers College of Nursing

Issue Number 23, Revised 2019

Editor-in-Chief: Sherry A. Greenberg, PhD, RN, GNP-BC
Managing Editor: Robin Coyne, MSN, RN, AGACNP-BC
New York University Rory Meyers College of Nursing

The Lawton Instrumental Activities of Daily Living (IADL) Scale

By: Robin Coyne, MSN, RN, AGACNP-BC, Wolters Kluwer

WHY: The assessment of functional status is critical when caring for older adults. Normal changes due to aging, acute illness, worsening chronic illness, and hospitalization can contribute to a decline in the ability to perform tasks necessary to live independently in the community. The information from a functional assessment can provide objective data to assist with targeting individualized rehabilitation needs or to plan for specific in home services such as meal preparation, nursing and personal care, home-maker services, financial and medication management, and/or continuous supervision. A functional assessment can also guide the clinician to focus on the person's baseline capabilities, facilitating early recognition of changes that may signify a need either for additional resources or for a medical work-up (Greenberg & McCabe, 2018).

BEST TOOL: The Lawton Instrumental Activities of Daily Living Scale (IADL) is an appropriate instrument to assess independent living skills (Lawton & Brody, 1969). These skills are considered more complex than the basic activities of daily living as measured by the Katz Index of ADLs (See *Try This:®* Katz Index of ADLs). The instrument is most useful for identifying how a person is functioning at the present time and for identifying improvement or deterioration over time. There are 8 domains of function measured with the Lawton IADL scale. Historically, women were scored on all 8 areas of function; men were not scored in the domains of food preparation, housekeeping, laundering. However, current recommendations are to assess all domains for both genders (Lawton, Moss, Fulcomer, & Kleban, 2003). Persons are scored according to their highest level of functioning in that category. A summary score ranges from 0 (low function, dependent) to 8 (high function, independent).

TARGET POPULATION: This instrument is intended to be used among older adults, and may be used in community, clinic, or hospital settings. The instrument is not useful for institutionalized older adults. It may be used as a baseline assessment tool and to compare baseline function to periodic assessments.

VALIDITY AND RELIABILITY: Few studies have been performed to test the Lawton IADL scale psychometric properties. The Lawton IADL Scale was originally tested concurrently with the Physical Self-Maintenance Scale (PSMS). Reliability was established with twelve subjects interviewed by one interviewer with the second rater present but not participating in the interview process. Inter-rater reliability was established at 0.85. The validity of the Lawton IADL was tested by determining the correlation of the Lawton IADL with four scales that measured domains of functional status, the Physical Classification (6-point rating of physical health), Mental Status Questionnaire (10-point test of orientation and memory), Behavior and Adjustment rating scales (4-6-point measure of intellectual, person, behavioral and social adjustment), and the PSMS (6-item ADLs). A total of 180 research subjects participated in the study, however, few received all five evaluations. All correlations were significant at the 0.01 or 0.05 level. To avoid potential gender bias at the time the instrument was developed, specific items were omitted for men. This assessment instrument is widely used both in research and clinical practice.

STRENGTHS AND LIMITATIONS: The Lawton IADL is an easy to administer assessment instrument that provides self-reported information about functional skills necessary to live in the community. Administration time is 10-15 minutes. Specific deficits identified can assist nurses and other disciplines in planning for safe hospital discharge.

A limitation of the instrument includes the self-report or surrogate report method of administration rather than a demonstration of the functional task. This may lead either to over-estimation or under-estimation of ability. In addition, the instrument may not be sensitive to small, incremental changes in function.

FOLLOW-UP: The identification of new disabilities in these functional domains warrants intervention and further assessment to prevent ongoing decline and to promote safe living conditions for older adults. If using the Lawton IADL tool with an acute hospitalization, nurses should communicate any deficits to the physicians and social workers/case managers for appropriate discharge planning.

MORE ON THE TOPIC:

Best practice information on care of older adults: https://consultgeri.org.
Graf, C. (2006). Functional decline in hospitalized older adults. *AJN, 106*(1), 58-67.
Graf, C. (2008). The Lawton Instrumental Activities of Daily Living Scale. *AJN, 108*(4), 52-62.
Greenberg, S. A., & McCabe, D. (2018). Functional assessment of older adults. In T. Fulmer, & B. Chernof (Eds.), *Handbook of Geriatric Assessment* (5th ed., pp. 231-240). MA: Jones and Bartlett Learning.
Lawton, M.P., & Brody, E.M. (1969). Assessment of older people: Self-maintaining and instrumental activities of daily living. *The Gerontologist, 9*(3), 179-186.
Lawton, M.P., Moss, M., Fulcomer, M., & Kleban, M. H. (2003). *Multi-level assessment instrument manual for full-length MAI.* North Wales PA: Polisher Research Institute, Madlyn and Leonard Abramson Center for Jewish Life.
Pearson, V. (2000). Assessment of function. In R. Kane, & R. Kane (Eds.), *Assessing Older Persons. Measures, Meaning and Practical Applications* (pp. 17-48). New York: Oxford University Press.

Permission is hereby granted to reproduce, post, download, and/or distribute, this material in its *entirety* for not-for-profit educational purposes only, provided that The Hartford Institute for Geriatric Nursing, New York University, Rory Meyers College of Nursing is cited as the source. This material may be downloaded and/or distributed in electronic format, including PDA format. Available on the internet at **www.ConsultGeri.org**. E-mail notification of usage to: **nursing.hign@nyu.edu.**

The Lawton Instrumental Activities of Daily Living Scale

A. Ability to Use Telephone

1. Operates telephone on own initiative; looks up and dials numbers 1
2. Dials a few well-known numbers 1
3. Answers telephone, but does not dial 1
4. Does not use telephone at all 0

B. Shopping

1. Takes care of all shopping needs independently 1
2. Shops independently for small purchases 0
3. Needs to be accompanied on any shopping trip 0
4. Completely unable to shop 0

C. Food Preparation

1. Plans, prepares, and serves adequate meals independently 1
2. Prepares adequate meals if supplied with ingredients 0
3. Heats and serves prepared meals or prepares meals but does not maintain adequate diet 0
4. Needs to have meals prepared and served 0

D. Housekeeping

1. Maintains house alone with occasion assistance (heavy work) 1
2. Performs light daily tasks such as dishwashing, bed making 1
3. Performs light daily tasks, but cannot maintain acceptable level of cleanliness 1
4. Needs help with all home maintenance tasks 1
5. Does not participate in any housekeeping tasks 0

E. Laundry

1. Does personal laundry completely 1
2. Launders small items, rinses socks, stockings, etc 1
3. All laundry must be done by others 0

F. Mode of Transportation

1. Travels independently on public transportation or drives own car 1
2. Arranges own travel via taxi, but does not otherwise use public transportation 1
3. Travels on public transportation when assisted or accompanied by another 1
4. Travel limited to taxi or automobile with assistance of another 0
5. Does not travel at all 0

G. Responsibility for Own Medications

1. Is responsible for taking medication in correct dosages at correct time 1
2. Takes responsibility if medication is prepared in advance in separate dosages 0
3. Is not capable of dispensing own medication 0

H. Ability to Handle Finances

1. Manages financial matters independently (budgets, writes checks, pays rent and bills, goes to bank); collects and keeps track of income 1
2. Manages day-to-day purchases, but needs help with banking, major purchases, etc 1
3. Incapable of handling money 0

Scoring: For each category, circle the item description that most closely resembles the client's highest functional level (either 0 or 1).

Lawton, M.P., & Brody, E.M. (1969). Assessment of older people: Self-maintaining and instrumental activities of daily living. *The Gerontologist*, 9(3), 179-186.

Copyright © The Gerontological Society of America. Reproduced [Adapted] by permission of the publisher.

The Hartford Institute for Geriatric Nursing recognizes Carla Graf as the original author of this issue.
