# Supplementary material for: Aerobic Exercise in HIV-Associated Neurocognitive Disorders: Protocol for a Randomized Controlled Trial
Source: JMIR Res Protoc. 2022 Jan 31;11(1):e29230. doi: 10.2196/29230 (PMC8844984; doi:10.2196/29230)
Supplement: Multimedia Appendix 10 [file resprot_v11i1e29230_app10.pdf]

THE WORLD HEALTH ORGANIZATION

QUALITY OF LIFE (WHOQOL) -BREF

The World Health Organization Quality of Life (WHOQOL)-BREF

© World Health Organization 2004

All rights reserved. Publications of the World Health Organization can be obtained from Marketing and Dissemination, World Health Organization, 20 Avenue Appia, 1211 Geneva 27, Switzerland (tel: +41 22 791 2476; fax: +41 22 791 4857; email: bookorders@who.int). Requests for permission to reproduce or translate WHO publications—whether for sale or for noncommercial distribution—should be addressed to Publications, at the above address (fax: +41 22 791 4806; email: permissions@who.int).

The designations employed and the presentation of the material in this publication do not imply the expression of any opinion whatsoever on the part of the World Health Organization concerning the legal status of any country, territory, city or area or of its authorities, or concerning the delimitation of its frontiers or boundaries. Dotted lines on maps represent approximate border lines for which there may not yet be full agreement.

The mention of specific companies or of certain manufacturers' products does not imply that they are endorsed or recommended by the World Health Organization in preference to others of a similar nature that are not mentioned. Errors and omissions excepted, the names of proprietary products are distinguished by initial capital letters.

The World Health Organization does not warrant that the information contained in this publication is complete and correct and shall not be liable for any damages incurred as a result of its use.

WHOQOL-BREF

The following questions ask how you feel about your quality of life, health, or other areas of your life. I will read out each question to you, along with the response options. **Please choose the answer that appears most appropriate.** If you are unsure about which response to give to a question, the first response you think of is often the best one.

Please keep in mind your standards, hopes, pleasures and concerns. We ask that you think about your life **in the last four weeks.**

		Very poor	Poor	Neither poor nor good	Good	Very good
1.	How would you rate your quality of life?	1	2	3	4	5

		Very dissatisfied	Dissatisfied	Neither satisfied nor dissatisfied	Satisfied	Very satisfied
2.	How satisfied are you with your health?	1	2	3	4	5

The following questions ask about **how much** you have experienced certain things in the last four weeks.

		Not at all	A little	A moderate amount	Very much	An extreme amount
3.	To what extent do you feel that physical pain prevents you from doing what you need to do?	5	4	3	2	1
4.	How much do you need any medical treatment to function in your daily life?	5	4	3	2	1
5.	How much do you enjoy life?	1	2	3	4	5
6.	To what extent do you feel your life to be meaningful?	1	2	3	4	5

		Not at all	A little	A moderate amount	Very much	Extremely
7.	How well are you able to concentrate?	1	2	3	4	5
8.	How safe do you feel in your daily life?	1	2	3	4	5
9.	How healthy is your physical environment?	1	2	3	4	5

The following questions ask about how completely you experience or were able to do certain things in the last four weeks.

		Not at all	A little	Moderately	Mostly	Completely
10.	Do you have enough energy for everyday life?	1	2	3	4	5
11.	Are you able to accept your bodily appearance?	1	2	3	4	5
12.	Have you enough money to meet your needs?	1	2	3	4	5
13.	How available to you is the information that you need in your day-to-day life?	1	2	3	4	5
14.	To what extent do you have the opportunity for leisure activities?	1	2	3	4	5

		Very poor	Poor	Neither poor nor good	Good	Very good
15.	How well are you able to get around?	1	2	3	4	5

		Very dissatisfied	Dissatisfied	Neither satisfied nor dissatisfied	Satisfied	Very satisfied
16.	How satisfied are you with your sleep?	1	2	3	4	5
17.	How satisfied are you with your ability to perform your daily living activities?	1	2	3	4	5
18.	How satisfied are you with your capacity for work?	1	2	3	4	5
19.	How satisfied are you with yourself?	1	2	3	4	5

20.	How satisfied are you with your personal relationships?	1	2	3	4	5
21.	How satisfied are you with your sex life?	1	2	3	4	5
22.	How satisfied are you with the support you get from your friends?	1	2	3	4	5
23.	How satisfied are you with the conditions of your living place?	1	2	3	4	5
24.	How satisfied are you with your access to health services?	1	2	3	4	5
25.	How satisfied are you with your transport?	1	2	3	4	5

The following question refers to how often you have felt or experienced certain things in the last four weeks.

		Never	Seldom	Quite often	Very often	Always
26.	How often do you have negative feelings such as blue mood, despair, anxiety, depression?	5	4	3	2	1

Do you have any comments about the assessment?

____

____

[The following table should be completed after the interview is finished]

		Equations for computing domain scores	Raw score	Transformed scores*	
				4-20	0-100
27.	**Domain 1**	(6-Q3) + (6-Q4) + Q10 + Q15 + Q16 + Q17 + Q18<br>☐ + ☐ + ☐ + ☐ + ☐ + ☐ + ☐	a. =	b:	c:
28.	**Domain 2**	Q5 + Q6 + Q7 + Q11 + Q19 + (6-Q26)<br>☐ + ☐ + ☐ + ☐ + ☐ + ☐	a. =	b:	c:
29.	**Domain 3**	Q20 + Q21 + Q22<br>☐ + ☐ + ☐	a. =	b:	c:
30.	**Domain 4**	Q8 + Q9 + Q12 + Q13 + Q14 + Q23 + Q24 + Q25<br>☐ + ☐ + ☐ + ☐ + ☐ + ☐ + ☐ + ☐	a. =	b:	c:

* See Procedures Manual, pages 13-15
