# Supplementary material for: Aerobic Exercise in HIV-Associated Neurocognitive Disorders: Protocol for a Randomized Controlled Trial
Source: JMIR Res Protoc. 2022 Jan 31;11(1):e29230. doi: 10.2196/29230 (PMC8844984; doi:10.2196/29230)
Supplement: Multimedia Appendix 11 [file resprot_v11i1e29230_app11.pdf]

Oxford Participation and Activities Questionnaire (Ox-PAQ)

How often during the ***past 4 weeks***
have you had difficulties with the following:

Please ***tick one box*** *for each question*

		Never	Rarely	Sometimes	Often	Always
**1.**	Getting up in the morning?	☐	☐	☐	☐	☐
**2.**	Getting dressed?	☐	☐	☐	☐	☐
**3.**	Getting around your home?	☐	☐	☐	☐	☐
**4.**	Doing household chores (e.g. cleaning your home, doing the laundry, cooking, taking out the rubbish)?	☐	☐	☐	☐	☐
**5.**	Going to the shops for everyday goods, such as food or household items?	☐	☐	☐	☐	☐
**6.**	Doing daily activities you would like to do?	☐	☐	☐	☐	☐
**7.**	Doing work, paid or unpaid?	☐	☐	☐	☐	☐
**8.**	Your social life (e.g. meeting friends or family)?	☐	☐	☐	☐	☐

Please check that you have ***ticked one box for each question***
before going onto the next page.

SAMPLE COPY

Ox-PAQ © Copyright, Oxford University Innovation Limited 2015. All Rights Reserved.
The authors, being Professors Crispin Jenkinson, Ray Fitzpatrick, Jill Dawson, Dr David Morley, Dr Laura Kelly and Mrs Sarah Dummett, have asserted their moral rights.

*How often during the **past 4 weeks***
have you had difficulties with the following:

*Please **tick one box** for each question*

		Never	Rarely	Sometimes	Often	Always
**9.**	Doing leisure activities that you enjoy (e.g. reading a book, listening to music, going out for a drink/meal)?	☐	☐	☐	☐	☐
**10.**	Doing physical activities that you enjoy (e.g. walking, gardening, swimming)?	☐	☐	☐	☐	☐
**11.**	Maintaining close relationships (e.g. with your partner/spouse, caregiver, parents, children)?	☐	☐	☐	☐	☐
**12.**	Maintaining friendships?	☐	☐	☐	☐	☐
**13.**	Engaging with people when out in the community (e.g. when shopping, at the bank)?	☐	☐	☐	☐	☐
**14.**	Being as independent as you would like?	☐	☐	☐	☐	☐
**15.**	Engaging in community life (e.g. voluntary activities, local clubs / groups)?	☐	☐	☐	☐	☐
**16.**	Using your own transport (e.g. bicycle, motorbike, car, mobility scooter)?	☐	☐	☐	☐	☐

SAMPLE COPY

Please check that you have ***ticked one box for each question***
before going onto the next page.

Ox-PAQ © Copyright, Oxford University Innovation Limited 2015. All Rights Reserved.
The authors, being Professors Crispin Jenkinson, Ray Fitzpatrick, Jill Dawson, Dr David Morley, Dr Laura Kelly and Mrs Sarah Dummett, have asserted their moral rights.

*How often during the **past 4 weeks** have you had difficulties with the following:*

*Please **tick one box** for each question*

	Never	Rarely	Sometimes	Often	Always
**17.** Using public transport (e.g. taxi, bus, train, tram)?	☐	☐	☐	☐	☐
**18.** Communicating successfully with other people?	☐	☐	☐	☐	☐
**19.** Feeling that you don't have control of your life?	☐	☐	☐	☐	☐
**20.** Feeling stressed?	☐	☐	☐	☐	☐
**21.** Feeling anxious?	☐	☐	☐	☐	☐
**22.** Feeling sad?	☐	☐	☐	☐	☐
**23.** Feeling depressed?	☐	☐	☐	☐	☐

SAMPLE COPY

Please check that you have ***ticked one box for each question***.

Thank you for completing the questionnaire.

Ox-PAQ © Copyright, Oxford University Innovation Limited 2015. All Rights Reserved.
The authors, being Professors Crispin Jenkinson, Ray Fitzpatrick, Jill Dawson, Dr David Morley, Dr Laura Kelly and Mrs Sarah Dummett, have asserted their moral rights.
