# Supplementary material for: Aerobic Exercise in HIV-Associated Neurocognitive Disorders: Protocol for a Randomized Controlled Trial
Source: JMIR Res Protoc. 2022 Jan 31;11(1):e29230. doi: 10.2196/29230 (PMC8844984; doi:10.2196/29230)
Supplement: Multimedia Appendix 12 [file resprot_v11i1e29230_app12.pdf]

Data Collection Sheet

NAME:______________________________________ DATE:________________

HEIGHT:_________in. WEIGHT:___________lbs. AGE:__________

PHYSICIANS NAME:__________________________ PHONE:_____________

PHYSICAL ACTIVITY READINESS QUESTIONNAIRE (PAR-Q)

	Questions	Yes	No
1	Has your doctor ever said that you have a heart condition and that you should only perform physical activity recommended by a doctor?		
2	Do you feel pain in your chest when you perform physical activity?		
3	In the past month, have you had chest pain when you were not performing any physical activity?		
4	Do you lose your balance because of dizziness or do you ever lose consciousness?		
5	Do you have a bone or joint problem that could be made worse by a change in your physical activity?		
6	Is your doctor currently prescribing any medication for your blood pressure or for a heart condition?		
7	Do you know of any other reason why you should not engage in physical activity?		

If you have answered "Yes" to one or more of the above questions, consult your physician before engaging in physical activity. Tell your physician which questions you answered "Yes" to. After a medical evaluation, seek advice from your physician on what type of activity is suitable for your current condition.

GENERAL & MEDICAL QUESTIONNAIRE

	Occupational Questions	Yes	No
1	What is your current occupation? ________________________________		
2	Does your occupation require extended periods of sitting?		
3	Does your occupation require extended periods of repetitive movements? (If yes, please explain.) ________________________________		
4	Does your occupation require you to wear shoes with a heel (dress shoes)?		
5	Does your occupation cause you anxiety (mental stress)?		
	**Recreational Questions**	**Yes**	**No**
6	Do you partake in any recreational activities (golf, tennis, skiing, etc.)? (If yes, please explain.) ________________________________ ________________________________		
7	Do you have any hobbies (reading, gardening, working on cars, exploring the Internet, etc.)? (If yes, please explain.) ________________________________ ________________________________		
	**Medical Questions**	**Yes**	**No**
8	Have you ever had any pain or injuries (ankle, knee, hip, back, shoulder, etc.)? (If yes, please explain.) ________________________________ ________________________________		
9	Have you ever had any surgeries? (If yes, please explain.) ________________________________ ________________________________		
10	Has a medical doctor ever diagnosed you with a chronic disease, such as coronary heart disease, coronary artery disease, hypertension (high blood pressure), high cholesterol or diabetes? (If yes, please explain.) ________________________________ ________________________________		
11	Are you currently taking any medication? (If yes, please list.) ________________________________ ________________________________ ________________________________		
