# Supplementary material for: Aerobic Exercise in HIV-Associated Neurocognitive Disorders: Protocol for a Randomized Controlled Trial
Source: JMIR Res Protoc. 2022 Jan 31;11(1):e29230. doi: 10.2196/29230 (PMC8844984; doi:10.2196/29230)
Supplement: Multimedia Appendix 13 [file resprot_v11i1e29230_app13.docx]

**Adverse Events Form**

Title of study: Effects of a 12-week Aerobic Exercise Programme on HIV-associated Neurocognitive Disorder

Investigator: MC Nweke Subject’s ID: Date of report:

- **Name of event Description of events/comments**

1. Exercise induced tachycardia? YES NO

2. Dyspnea? YES NO

3. Pain? YES NO

4. Fatigue or muscle soreness? YES NO

5. bursitis? YES NO

6. low back pain? YES NO

7. oedema? YES NO

8. Hospitalization? YES NO

9. Stroke? YES NO

10. Fracture? YES NO

- **If YES to any of the adverse event listed above**

11. Date of event's onset? ____________

12. Date of resolution? _______________

13. Intensity? mild moderate or severe

14. Relationship to exercise? probably related possibly related not related

15. Anticipated? YES NO

16. Treatment given, if any _______________________________________________
